# Exchanging ligand-binding specificity between a pair of mouse olfactory receptor paralogs reveals odorant recognition principles

**DOI:** 10.1038/srep14948

**Published:** 2015-10-09

**Authors:** Olivia Baud, Shuguang Yuan, Luc Veya, Slawomir Filipek, Horst Vogel, Horst Pick

**Affiliations:** 1 Institut des Sciences et Ingénierie Chimiques, École Polytechnique Fédérale de Lausanne, CH-1015 Lausanne Switzerland; 2 Faculty of Chemistry, Biological and Chemical Research Centre, University of Warsaw, Poland

## Abstract

A multi-gene family of ~1000 G protein-coupled olfactory receptors (ORs) constitutes the molecular basis of mammalian olfaction. Due to the lack of structural data its remarkable capacity to detect and discriminate thousands of odorants remains poorly understood on the structural level of the receptor. Using site-directed mutagenesis we transferred ligand specificity between two functionally related ORs and thereby revealed amino acid residues of central importance for odorant recognition and discrimination of the two receptors. By exchanging two of three residues, differing at equivalent positions of the putative odorant binding site between the mouse OR paralogs Olfr73 (mOR-EG) and Olfr74 (mOR-EV), we selectively changed ligand preference but remarkably also signaling activation strength in both ORs. Computer modeling proposed structural details at atomic resolution how the very same odorant molecule might interact with different contact residues to induce different functional responses in two related receptors. Our findings provide a mechanistic explanation of how the olfactory system distinguishes different molecular aspects of a given odorant molecule, and unravel important molecular details of the combinatorial encoding of odorant identity at the OR level.

The mammalian olfactory system employs a large gene family of up to more than 1000 G protein-coupled odorant receptors (OR) to detect and to distinguish a nearly unlimited number of chemically diverse odorous compounds^1^,^2^,^3^,^4^. These are typically small organic molecules of less than 300 Da and highly variant chemical structures^5^,^6^,^7^. The binding of odorants to their ORs located on the cilia of specialized olfactory sensory neurons (OSN) in the nasal, olfactory epithelium represents the primary event of odor detection initiating a neuronal response that triggers the perception of a smell in the brain^8^,^9^.

A few millions of OSNs constitute the peripheral olfactory system and each OSN expresses only a single type of receptor out of the 1000 members of the OR family^10^. For achieving the enormous discriminatory power of recognizing many thousands of compounds as distinct odors it is assumed that individual ORs recognize multiple odorants and that the receptors are used in a combinatorial manner to distinguish specific odorant molecules^11^. To date there is only little knowledge about the ligand specificity of the majority of mammalian ORs. This is mostly due to difficulties in achieving functional expression of these proteins in heterologous cells^12^. Cognate ligands identified sofar for a limited number of ORs indicate that each receptor recognizes an array of odorants^13^,^14^,^15^,^16^,^17^,^18^.

There is increasing evidence that ORs recognize their cognate ligands by a network of distinct amino acid residues disposed in their trans-membrane domains (TM). Specific residues in TM3, TM5 and TM6 were experimentally validated to define odorant specificity of a number of different ORs^18^,^19^,^20^. These residues often overlap with evolutionally conserved ligand contact sites, which have previously been proposed by comparisons of mouse-human OR orthologs^21^. However, additional residues apart from that generalized odorant-binding pocket may also be functionally involved in odorant recognition^18^,^19^,^22^.

Overall, the structural base of small molecule recognition and discrimination in mammalian olfaction remains largely unexplored, as crystal structures of ORs have not been determined yet. Under these circumstances progress in understanding the receptor recognition of odorants can be made by combining less direct but complementary approaches such as studying how odorants of different chemical structures might activate the same receptor or how point mutations in the receptor can influence the activation profile of odorants which finally deliver a solid data base for building 3D structural homology models to crystal structures of related GPCRs.

With the assumption that ORs of high coding sequence homology may have similar functions we performed a detailed comparative analysis of a pair of paralogous ORs, the mouse eugenol receptor Olfr73 (mOR-EG) and the mouse ethyl-vanillin receptor Olfr74 (mOR-EV) in order to identify molecular determinants of odorant recognition. The two ORs display 79% overall identity on amino acid sequence level. An even higher sequence identity of 86% is pronounced in their transmembrane helical domains, which are supposed to form the putative ligand-binding domain^23^. Paralogous ORs have diverged in evolution from a common ancestral gene^21^. The evolutionary distinct but related properties of such receptors are important natural sources for building rational models of the receptors’ odorant binding sites and to reveal structure-function relationships of OR proteins.

In a previous study we identified in Olfr73 eleven amino acids as an essential part of the receptor’s ligand binding site located in TM3, TM5 and TM6 and including the second extracellular loop^18^. Six of these residues overlap with a proposed generalized OR binding pocket^21^. Precise odorant interactions sites of Olfr74 have not been analysed to date. We and others have screened compound libraries^18^,^19^ revealing 38 distinct odorants capable of activating Olfr73. Less is known about the molecular receptive range of its paralog Olfr74. Thus far the odorants vanillin and ethyl vanillin were found to activate both Olfr74 (mOR-EV) and Olfr 73 (mOR-EG)^23^.

Here we report the activating ligand spectrum of Olfr74. Screening a library of 270 compounds we have found jasmone as a new potent agonist specific only to Olfr74 and two less potent odorants, rose oxide and nootkatone, activating both paralogous ORs with different efficacies. Thus, screening the same library of odorant compounds on both receptors revealed striking differences in the broadness of their molecular receptive ranges. Whereas Olfr73 responds to many odorants of diverse chemical structure, Olfr74 accepts just a few structurally different odorants. The pronounced difference in ligand specificity between the two paralogs in spite of their substantial high sequence identity prompted us to introduce mutations in both receptors that exchanged their ligand preferences. In order to explain the results of our functional assays on a structural level by localizing TM residues, which might define odorant specificity in Olfr73 and Olfr74 we used homology modelling and molecular dynamics (MD) simulations based on the atomic structure of the activated β-2 adrenergic receptor^24^. Progress in structural biology of GPCRs made it possible to provide by computer modelling reliable propositions of central steps in GPCR mediated signalling, including odorant and taste receptors which are difficult or impossible to obtain only by experiments^16^,^19^,^22^,^25^,^26^,^27^,^28^. Our approach to combine functional mutational analysis with molecular modelling delivered a reliable explanation of odorant-receptor interactions at atomic resolution and in turn a reasonable proposal of ligand-induced activation mechanisms in Olfr73 and Olfr74. Revealing such key molecular features of odorant-receptor interactions is of central importance for the basic understanding of odorant coding at the receptor level, for predictions and rational design of activating ligands on the specific level of ORs and G protein-coupled receptors (GPCRs) in general.

## Results and Discussion

**Figure 1:**
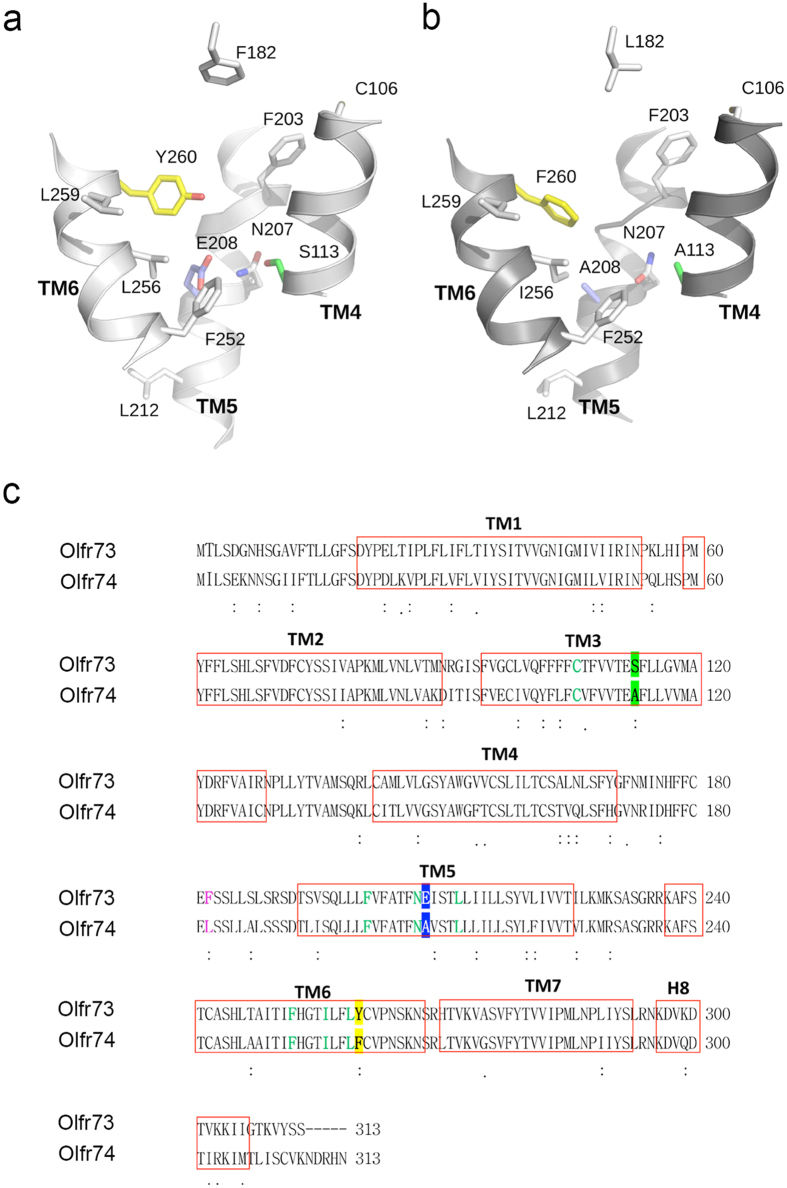
Predicted 3D models of Olfr73 and Olfr74 odorant binding pockets. (**a**) Experimentally confirmed amino acid residues functionally relevant for odorant detection in Olfr73: C106 (TM3), S113 (TM3), F182 external loop (EL2), F203 (TM5), N207 (TM5), E208 (TM5), L212 (TM5), F252 (TM6), I256 (TM6), L259 (TM6), Y260 (TM6) are compared with homologous amino acid positions in Olfr74 (**b**). Different amino acids in positions 113, 208 and 260 of both paralogs are indicated with green, blue and yellow colors, respectively. (**c**) Sequence alignment between Olfr73 and Olfr74. Residues functionally important for odorant detection in Olfr73 and identical in Olfr74 are shown in mint color, those that differ are shown in green, blue and yellow, respectively. Different amino acids in position 182 (EL2) are shown in violet. The putative TMs are *boxed in red*.

Among the cluster of 11 residues, which we previously identified to be important for odorant recognition in Olfr73, the amino acids S113 (TM3), E208 (TM5) and Y260 (TM6) serve as hydrogen donors in the central part of its ligand-binding pocket (Fig. 1a)^18^. Sequence alignment between Olfr73 and Olfr74 revealed that functionally important odorant interaction sites in both paralogs are identical except for the three respective core positions where different residues A113, A208, F260 can be found in Olfr74 (Fig. 1b).

To analyse structural determinants of odorant recognition in Olfr73 and Olfr74, we focused on the following questions: Are the key amino acid positions 113, 208, and 260 implicated in modulating odorant specificity in both receptors? Is it possible to swap ligand specificity from Olfr74 to Olfr73 by exchanging the predicted odorant interacting residues from one to the other receptor?

In order to minimize the complexity of our analysis the difference at another amino acid position (F182, Olfr73;L182, Olfr74) located in the second extracellular loop (ECL2) was not taken into account (Fig. 1c).

Before entering into molecular investigations of odorant binding and receptor activation we first established the expression and membrane targeting of Olfr74 in Hana3A cells^29^,^30^. Plasma membrane localization of the receptor was confirmed by immunocytochemistry, which is prerequisite of its functional expression (Supplementary Fig. 1). Hana3A cells allow coupling of OR activation to G_αolf_ and the cyclic AMP second messenger pathway^29^,^30^. Following this approach we had determined the ligand spectrum of Olfr73 in a previous study^18^,^19^. Here we used the same strategy and compound library to screen for Olfr74 activating odorants.

### Odorant response profiles of Olfr73 and Olfr74

**Figure 2:**
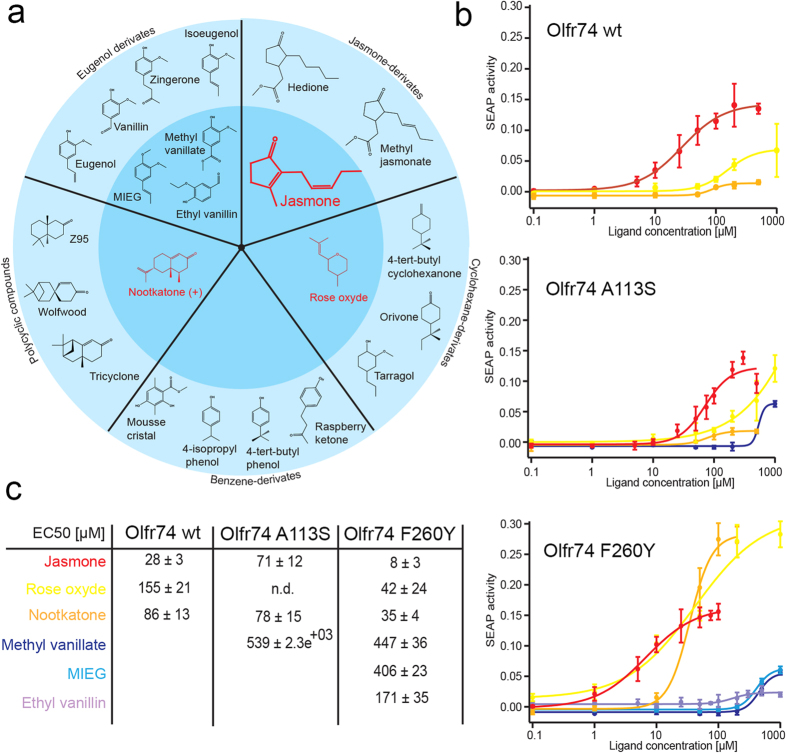
Overview of Olfr74 activating odorants. (**a**) Chemical structures of the Olfr74-specific agonist jasmone and its derivatives as well as structures of four Olfr73-specific classes of odorants: eugenol-, cyclohexane-, benzene derivatives, and polycyclic compounds.Odorants activating Olfr74 wt (red) or Olfr74 point mutants (in black) are grouped in the middle dark blue circle. Those with no detectable activity are shown in the external light blue circle. (**b**) Dose response profiles of odorants activating Olfr74 wild type and mutants. (**c**) EC_50_ values (μM) of different OR activating compounds.

Screening Olfr74 against 270 odorants revealed jasmone (3-methyl-2-(pent-2-enyl)-cyclopent-2-enone) as a novel and potent agonist with an EC_50_ value of 28 ± 3 μM (Fig. 2). Furthermore, two Olfr73-specific odorants, the cyclohexane derivate rose oxide and the polycylic compound nootkatone, were also found to activate Olfr74 with an EC_50_ value of 155 ± 21 μM for rose oxide and 86 ± 13 μM for nootkatone (Fig. 2).

Ethyl vanillin was previously postulated as an agonist common to both Olfr73 and Olfr74, but only at elevated millimolar concentrations^23^. Using a cAMP-dependent reporter (secreted alkaline phosphatase, SEAP) we revealed activation of Olfr73 by ethyl-vanillin with an EC_50_ value of 35 ± 4 μM whereas no response was detected for Olfr74 (Supplementary Fig. 2). This discrepancy to former reports^23^ may be explained by the fact that in previous experiments the cDNA encoding the olfactory G protein G_αolf_ was transiently co-transfected at increasing amounts until Olfr74 signaling responses to ethyl vanillin were obtained^23^, whereas we used here the Hana3A cell line without manipulating its endogenous G_αolf_ expression level. Consistent with our results, elsewhere no cAMP signalling was found upon stimulation of Olfr74 by EV in HEK cells^31^. In our present study we only achieved activation of Olfr74 by ethyl vanillin after introducing the point mutation F260Y rendering its putative ligand-binding site sequence more similar to that of Olfr73 (Fig. 2, Supplementary Fig. 3).

Taken together, we have identified jasmone, rose oxide and nootkatone as novel Olfr74-specific agonists. While we, and others have described in total a spectrum of 38 Olfr73-specific ligands with diverse chemical structures^18^,^19^, we identified for its paralog Olfr74 only three, but structurally unrelated agonists. Our results indicate an overlapping ligand specificity of Olfr73 and Olfr74 only for two odorants nootkatone and rose oxide.

### Olfr74_F260Y_ confers a gain of function towards structurally diverse odorants

We tested the hypothesis, that OR ligand specificity might be determined by single, evolutionary conserved amino acid positions in the putative odorant binding pocket. We exchanged A113, A208 and F260 in Olfr74 into respective amino acids of Olfr73 and vice versa. One amino acid was either exchanged at a time or all three simultaneously, by introducing point mutations at the equivalent positions. Then we tested activation of these mutants towards a number of odorants specific to one or both receptors.

The odorants rose oxide and the nootkatone are less potent agonists for Olfr74 than jasmone (jasmone/rose oxide: p < 0.0000006, jasmone/nootkatone: p < 0.0001). However, the mutation F260Y significantly enhanced the activation response of Olfr74 for all three odorants. Dose response curves in Fig. 2 show significantly reduced EC_50_ values for Olfr74_F260Y_ as compared to Olfr74 wildtype. For rose oxide an EC_50_ of 42 μM (EC50_wt_ = 155 μM) (p < 0.000005), for nootkatone 35 μM (EC50_wt_ = 86 μM) (p < 0.001), and jasmone EC50_F260Y_ = 8 μM (EC50_wt_ = 28 μM) (p < 0.05) was obtained, respectively (Fig. 2b,c).

Interestingly, Olfr74_F260Y_ also resulted in a gain of function towards the three Olfr73-specific odorants: ethyl-vanillin, methyl isoeugenol (MIEG) and methyl-vanillate, which do not activate wild type mOR-EV. Here we show that Olfr74_F260Y_ specifically responded to these odorants in a concentration-dependent fashion, however with high EC_50_ values in the range of 170 μM–400 μM and low amplitudes of cAMP responses (Fig. 2b,c).

The mutation A113S, showed either a minor effect on Olfr74 responsiveness to nootkatone or slightly diminished responses to jasmone (Fig. 2b) (p < 0.0025). However, the single A208E mutation and also the triple mutation (A113S, A208E, F260Y) conferred a complete loss of function towards all tested odorants (Supplementary Fig. 3).

Taken together, a single point mutation (F260Y) in Olfr74, which increases the amino acid sequence similarity to Olfr73, yields a gain of function towards some Olfr73-specific ligands and significantly improves the response to some Olfr74-specific odorants.

### Olfr73_E208A_ confers a gain of function towards the Olfr74-specific odorant jasmone

The single point mutation E208A in Olfr73 was sufficient to elicit responses by the Olfr74-specific agonist jasmone, which exhibits no structural similarity to other mOR-EG activating odorants (Table. 1, Supplementary Fig. 4). The triple mutation (S113A, E208A, Y260F) showed a similar EC_50_ value as E208A for jasmone indicating that a further increase of sequence similarity between mOR-EV and mOR-EG in positions 113 and 260 did not confer any additional gain of function towards the activation by this odorant (Table 1, Supplementary Fig. 4).

Although the E208A point mutation Olfr73 slightly improved responsiveness to its cognate ligands rose oxide and methyl isoeugenol (MIEG) as indicated by decreased EC_50_ values, compared to the wild type Olfr73, these changes were not significant according to the two-tailed student’s t-test (Table 1, Supplementary Fig. 4). The Olfr73_E208A_ mutation had also no significant effect on the activation by nootkatone (Table 1, Supplementary Fig. 4). Whereas the E208A mutation did not significantly influence activation by non-polar odorants, it resulted in a significant loss of function towards the more polar odorant ethyl vanillin or (Table 1, Supplementary Fig. 4) (p < 0.00001). However, the E208A mutation had less pronounced and non-significant effects on the activation by the polar odorant methyl vanillate, exhibiting an EC_50_ = 347 μM as compared to EC_50_ = 240 μM of the wildtype Olfr73 (Table 1, Supplementary Fig. 4).

The point mutation Olfr73 S113A showed a slightly decreased EC_50_ value for its cognate ligand rose oxide (EC_50_ = 20 μM as compared to EC_50_ = 40 μM of wt Olfr73), which was however not significant according the two-tailed student’s t test. The point mutation Y260F and the triple mutation (S113A, E208A, Y260F) completely abolished activation by all tested odorant compounds (Table 1, Supplementary Fig. 4). Taken together, the point mutation in E208 has most pronounced effects in changing responsiveness of Olfr73 towards a new ligand specificity allowing its activation by the Olfr74-specific odorant jasmone.

We have chosen to compare the odorant-binding mode of Olfr73 and its paralog Olfr74 at an atomic level in order to identify structural determinants of odorant recognition and receptor activation. Our functional screenings have revealed jasmone as a novel potent agonist specific only to Olfr74 and overall low functional similarity between the two ORs.

We found that Olfr73 and Olfr74 despite of their high coding sequence identity (~80%) evidently developed divergent ligand specificities in evolution, as they have only two structurally non-related agonists nootkatone and rose oxide in common. Moreover these odorants are not equally potent agonists for both receptors. By contrast the human paralogs OR1A1 and OR1A2 share only 50% sequence identity but they have 18 overlapping agonists out of a panel of 94 odorants^22^. These findings suggest that a high level of OR sequence homology not necessarily correlates with high functional similarity. However, OR orthologs, which originate from the same ancestral gene that diverged since a speciation event respond in more than 80% of the cases to a common ligand, whereas paralogs, which are related by a gene duplication event, only in 30% of the cases as revealed by a multi-receptor functional comparison between rodents and primates using the same panel of odorants^32^.

**Figure 3:**
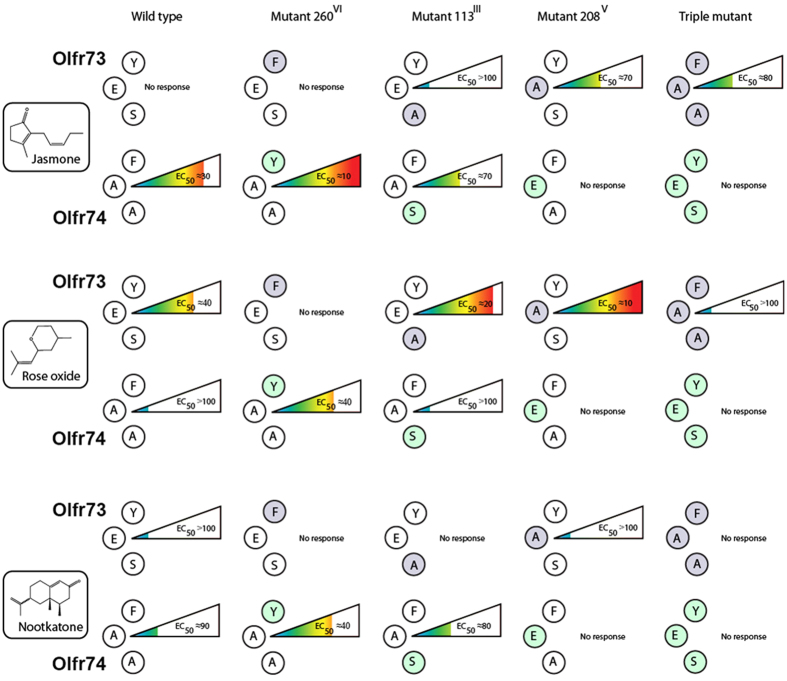
Potency of different odorants on Olfr73 and Olfr74 wildtype and mutant receptor activation. Schematic representation of the potency of jasmone, rose oxide and nootkatone in activating Olfr73, Olfr74 wildtype receptors and the corresponding point mutants in positions 113, 208 and 260. Specific amino acid changes are highlighted with grey and green shaded circles. Roman numbers indicate numbers of trans-membrane helices. The potency of OR activation is illustrated with a rainbow color spectrum ranging from blue corresponding to a low efficacy values (EC_50_ > 100 μM) to red corresponding to high efficacy values (EC_50_ = 10 μM). No response: no receptor activation detectable in the reporter assay. Corresponding dose response curves are shown in Supplementary Fig. 3 and Supplementary Fig. 4.

We discovered the functional relevance of the E208A mutation for altering the response behavior of Olfr73. This single point mutation resulted in a gain of function towards the Olfr74-specific odorant jasmone (Fig. 3). Regarding Olfr74 only the F260Y mutation conferred a significant gain of function towards a number of odorants, like jasmone (p < 0.05), rose oxide (p < 0.000005), and nootkatone (p < 0.001) (Fig. 3). In this way, our results provide direct insight into the nature of odorant-OR interactions and the functional role of specific amino acids in determining the receptive range of a receptor.

To date, only a few studies have examined changes in ligand selectivity in pairs of OR orthologs and paralogs on a molecular level. They demonstrate that single or double amino acid exchanges that differ at respective positions in the binding site of two closely related OR were sufficient to switch the ligand preference from heptanal versus octanal between two orthologs, and (R)-(+)-citronellol versus (S)-(−)-citronellol) between two paralogs, respectively^22^,^33^. This indicates the functional relevance of single amino acid positions or combinations of a few amino acids for the fine-tuning of ligand specificity regarding specific structural differences of closely related odorants. In our present study we have shown that single amino acid exchanges at positions relevant for odorant interactions can also be sufficient to confer specificity for unrelated odorant structures. Obviously changes not in the same but in two different amino acid positions 208 (TM5) in Olfr73 and 260 (TM6) in Olfr74, are responsible for altering their odorant responsiveness. This suggests the possibility of different binding-modes for the same specific ligands in the two mouse receptors.

To provide a reasonable explanation for the distinct odorant specificities of Olfr74 and Olfr73 on a protein structural basis, we have employed homology modeling of the ORs and molecular dynamics simulations using the structure of the activated β_2_-adrenergic receptor as a template^24^. This choice was based on the following considerations: (i) The β_2_-adrenergic receptor belongs to the same class A of GPCRs like ORs and rhodopsin, but is functionally more closely related to ORs than rhodopsin as it is responding to a freely diffusing ligand and is coupling to cyclic adenosine monophosphate (cAMP) second messenger pathway^34^,^35^. (ii) The active receptor conformation should provide the proper dynamic structural framework allowing for interactions of odorants with certain amino acid side chains. (iii) As most odorants show high structural flexibility, receptor-odorant molecular dynamics (MD) simulations are expected to provide important insights into receptor-odorant dynamics occurring during receptor activation, both on the receptor and on the ligand.

**Figure 4:**
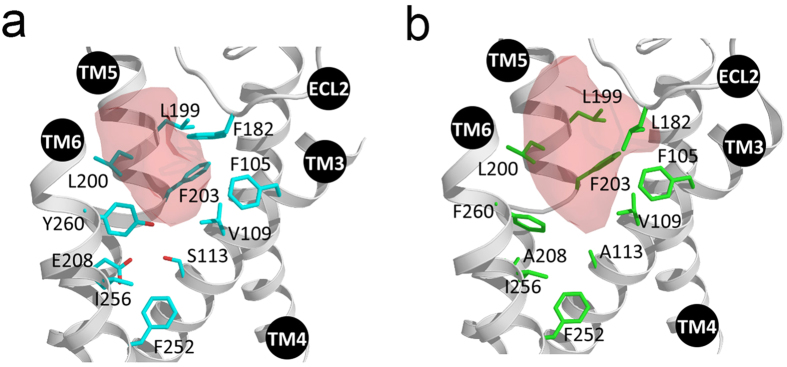
Comparison of binding cavity volumes of Olfr73 and Olfr74. Close-up view of 3D models showing the odorant binding cavities of (**a**) Olfr73 and (**b**) Olfr74. Residues are shown in blue and green. Numbers refer to the OR amino acid sequence. The binding cavity volumes are shown as red shaded areas.

Throughout MD simulations, the backbone structures of Olfr73 and Olfr74 models were quite stable showing no significant rearrangements (Supplementary Fig. 5). The side-by-side comparison of the 3D models (Fig. 4) indicates that the binding pockets of Olfr73 and Olfr74 comprise several identical hydrophobic residues close to their extracellular regions. However, the binding pocket of Olfr74 is visibly larger in size than that of Olfr73. This is mostly due to residues with small side chains present in several functionally relevant positions of Olfr74 versus Olfr73 (e.g. L182/F182; A113/S113; F260/Y260 and A208/E208). The ligand cavity size calculation by the Sitemap^36^ utility in Schroedinger suite further confirmed this hypothesis as we find a smaller ligand accessible volume for Olfr73 (200 Å^3^) than for Olfr74 (250 Å^3^). Such differences imply that Olfr74 potentially could bind bigger-sized ligands than Olfr73. In turn, as Olfr74 provides increased distances between ligand-binding contact sites it may prevent the formation of functionally active network of contact sites with many of the Olfr73 specific ligands. In addition the polar or charged amino acid residues of Olfr73, S113, E208 and Y260 have hydrophobic counterparts A113, A208, F260 in Olfr74 changing also the chemical properties between both binding sites. Thus, a number of different molecular and structural properties may explain why we find striking differences between the ligand activation profile of Olfr73 and Olfr74.

### The two mouse paralogs exhibit different contact sites for common odorants

Our point mutation analysis has revealed an essential role of the amino acid residue in position 260 for Olfr73 and Olfr74 activation. Whereas the mutation Y260F in Olfr73 inactivates the receptor, Olfr74_F260Y_ results in gain of Olfr73 like function (Fig. 3). Our molecular dynamic simulation studies, which will be detailed in the following, indicate that Y260 functions as a gate of OR activation.

**Figure 5:**
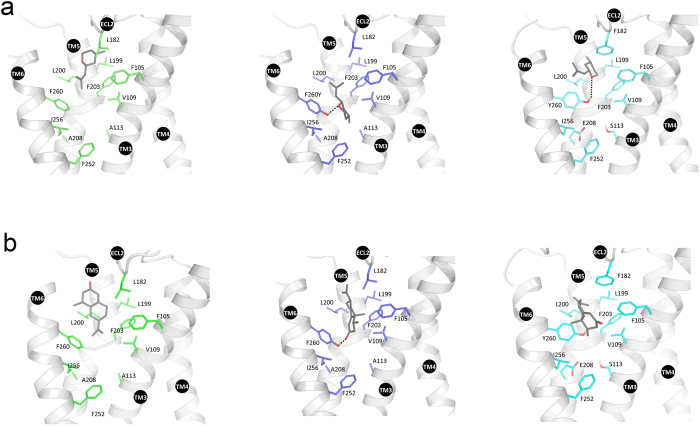
Rose oxide and nootkatone docked in the calculated Olfr73 and Olfr74 models. (**a**) left: interactions of Olfr74 (WT) with rose oxide; middle: rose oxide docked in Olfr74 (F260Y); right: rose oxide docked in Olfr73 (WT) (**b**) left: nootkatone docked in Olfr74 (WT); middle: nootkatone docked in Olfr74 (F260Y); right: nootkatone docked in Olfr73 (WT).

Docking poses of odorant-OR interactions indicate a different location for the same specific ligands in the Olfr73 and Olfr74 models with distinct impact on receptor activation. For instance rose oxide in the best docking position binds Olfr74 in a region toward the extracellular end of the receptor close to ECL2 (Fig. 5a, left panel). Its binding site is surrounded by hydrophobic residues: L199, L200, F203, F105, and L182 not making any contact with residues further down in the receptor such as F260, I256 or F252. Regarding position 260 as a “gate”, then rose oxide in its lowest energy pose is too far away to open it efficiently (Fig. 5a, left panel), which might well explain its weak potency on Olfr74 activation.

Conversely, in Olfr73 the binding pocket for rose oxide is located much deeper inside the receptor structure (Fig. 5a, right panel). Besides hydrophobic interactions with L199, L200, V109, F105 and F182, the ligand additionally forms hydrogen bonds with the oxygen of the side chain of Y260. In this docking position the “key” perfectly matches the “lock” thereby triggering the gate for receptor activation (Fig. 5a, right panel). This is consistent with our functional assays revealing rose oxide as a potent agonist of Olfr73. Interestingly, the mutation F260Y in Olfr74 can also open the gate and effectively activate the receptor by rose oxide. Here our computer modeling indicates that rose oxide is capable of forming hydrogen bonds with Y260 in Olfr74_F260Y_ quite similar as in Olfr73 (WT) (Fig. 5a, Supplementary Fig. 6).

Surprisingly, the triple mutation Olfr74_F260Y-A208E-A113S ,_ which further increases the sequence similarity to the Olfr73 binding-site exhibits no detectable activation by rose oxide. A reasonable explanation might be that the ligand in Olfr74_F260Y_ is already bound at a functionally active position to open the gate, and additional mutations (A208E; A113S) may alter the orientation of Y260 into a conformation unfavorable for activation through water mediated hydrogen bonding network as indicated in Supplementary Fig. 6.

The situation is quite different for nootkatone, which is a much bigger ligand than rose oxide, and therefore better fits into the larger binding pocket of Olfr74 than into Olfr73. This is consistent with our calculations indicating a restrained energy of 0.4 kcal/mol for the binding of nootkatone to Olfr74 as compared to 4.4 kcal/mol for the binding to Olfr73 (Supplementary Table 1). In our functional assays nootkatone was not a significantly weaker agonist for Olfr73 than for Olfr74. In Olfr74 nootkatone can be docked in a similar position of its odorant-binding site like rose oxide making no contacts to residues F260, I256 or F252 (Fig. 5b, left panel). In analogy to rose oxide the point mutation F260Y in Olfr74 leads also to an enhancement of receptor activation by nootkatone because of F260Y introduces additional hydrogen bonds for both agonists (Fig. 5a,b, middle panel), resulting in a higher binding affinity (Supplementary Table 1) and improved the EC_50_ values for both ligands. Nootkatone however is too big to be docked at the corresponding position in Olfr73 (Fig. 5b, right panel, Supplementary Fig. 6b, right panel). Its pose is shifted away from the center of the hydrophobic pocket to a zone nearby. The mutation E208A in Olfr73 results in a slight drop of the restrained energy but does not significantly improve the binding of nootkatone to the receptor and neither its EC_50_ value (Supplementary Table 1).

**Figure 6:**
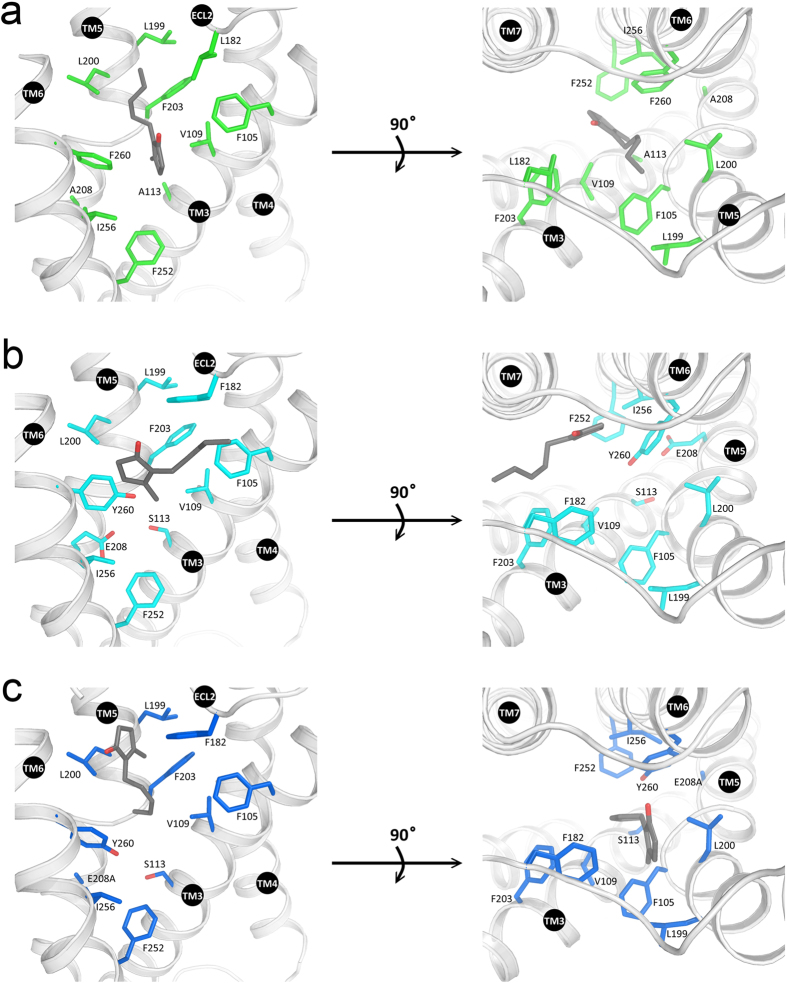
Jasmone docked in the calculated Olfr73 and Olfr74 models. (**a**) Jasmone (black stick) docked in wildtype Olfr74. (**b**) Jasmone docked in wildtyp Olfr73. (C) Jasmone docked in Olfr73 (E208A).

Jasmone is the most potent agonist of Olfr74 while it is inactive for Olfr73. Computer modeling shows that jasmone binds to Olfr74 in the center of a hydrophobic pocket consisting of L182, L199, L200, F203, V109 and F105 (Fig. 6a). Moreover, σ-π stacks were found between F260, jasmone and V109. By contrast, the location of jasmone in Olfr73 shifts from that region close to F105 to a zone near I256 and F182 due to steric problems (Fig. 6b). Only the E208A mutation in Olfr73 offers enough space for jasmone binding and receptor activation (Fig. 6c). Taken together jasmone activates both wildtype Olfr74 and Olfr73_E208A_ but we predict differences in the active binding pose for jasmone in the two receptors. Jasmone in Olfr74 can interact with F260 by its five membered ring leaving the alkane tail close to ECL2. By contrast jasmone in Olfr73_E208A_ interacts with Y260 by the alkane tail leaving the five-membered ring next to ECL2 (Fig. 6c). Moreover, the A208E mutation completely deactivates Olfr74 as E208 is close to the crucial residue F260 whose side chain conformation might be changed thereby. We find that the interactions sites for rose oxide, nootkatone and jasmone differ in both wildtype and mutated receptors.

### Predicted activation mechanism of Olfr73/ Olfr74

As another important finding our MD simulations have revealed a water molecule mediated extended hydrogen bonding network between Y260, E208 and S113 in Olfr73 (Supplementary Fig. 7). Such water pathways inside receptor structures likely play a general role in the trans-membrane signaling processes of GPCRs, as recently proposed^26^,^37^,^38^. Investigating the sequential steps of odorant-OR interactions and the subsequent conformational changes associated with receptor activation will be essential for improving the understanding of olfactory signaling at a molecular level. Thus, our present study is an important step towards this goal.

**Figure 7:**
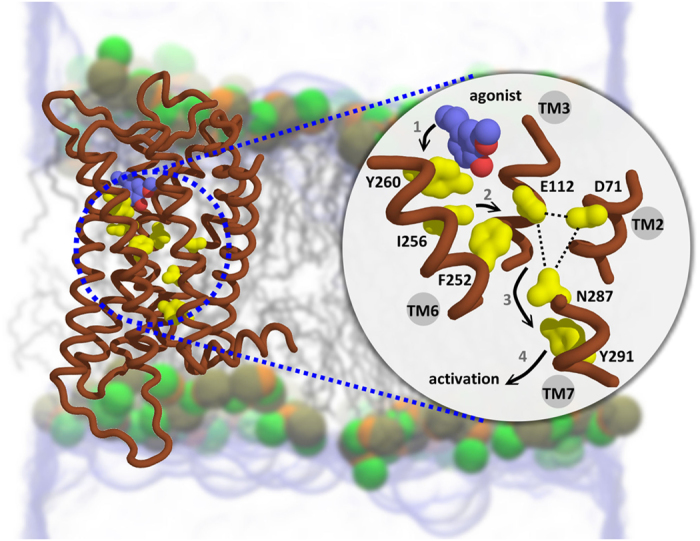
Proposed activation mechanism for Olfr73 and Olfr74. The binding of an agonist induces a molecular switch at position 260 (TM6) (step 1) which is further transferred to I256 and F252 along TM6 deeper into the receptor structure (step 2). Conformational fluctuations of F252 may have functional impact on the solvent mediated hydrogen bond network formed between E112 (TM3), D71 (TM2) (2.50) and N287 (TM7) (locates in NPxxY motif) (step 3). The interruption of this network can further lead to the breaking of hydrogen bonds between N287 (TM7) and Y291 (TM7). The switches of Y291 are coupled to the movement of TM7 to induce the dissociation of the G-protein, bound to Y291 (step 4).

Combining the functional mutagenesis data with molecular simulations on the new homology models of Olfr73/Olfr74 allowed us to propose a reasonable mechanism of how odorant binding induces conformational changes resulting in OR activation (Fig. 7). We have found that the two paralogs bind the same agonists in slightly different modes followed by the activation of a molecular switch at the equivalent position (residue 260) in both receptors. In Olfr73 agonist binding is driven by both hydrophobic interactions and a hydrogen bond formation at Y260; while in Olfr74 conformational switches mainly arise from hydrophobic interactions between agonist and the receptor. Different odorant binding modes (e.g for rose oxide, nootkatone) involve interactions with different amino acids in Olfr73 versus Olfr74. Docking of an odorant molecule into the OR binding cavity exerts stress to the protein structure leading to a sequence of conformational changes such as the molecular switch at position 260 followed by a movement of I256 and F252 deeper into the receptor. Fluctuations of the hydrophobic residue F252, as measured by MD simulations have substantial functional impact on solvent mediated hydrogen bond networks between residues E112 (TM3), D71 (TM2) (2.50) and N287 (TM7) (located in NPxxY motif), which are highly conserved residues in family A GPCRs. The interruption of this network can further lead to the breaking of hydrogen bonds between N287 (TM7) and Y291 (TM7). The conformational switch of Y291 might couple with the movement of TM7 leading to the dissociation of the G-protein, bound to Y291 nearby. Similar broken molecular interactions have been observed in the crystal structure of activated rhodopsin^39^.

In summary, we have identified critical amino acid residues determining the odorant specificity of Olfr73 and Olfr74. Point mutations conferring changes in the functional response of the receptors were those affecting either the binding cavity volume, or conformational changes in the receptor structure, occurring upon odorant binding. These observations indicate that although the OR binding cavity exhibits some degree of plasticity it is pre-disposed towards certain odorant sizes and structures. Interactions of odorants with multiple amino acid side chains may be variable and dynamic depending on the odorant structure and occur in a series of conformational intermediates with distinct functional properties as our molecular simulation studies indicate. This is consistent with recent findings that functionally distinct ligands of different structure induce multiple receptor conformations in the β_2_-adrenergic receptor underscoring the general importance of our present findings^40^. Hence we provide novel atomic-level insights regarding structure-activity relationships of odorants as well as structure-function relations of ORs.

Moreover, we have revealed different binding modes for particular odorants on two functionally related ORs implying that each receptor ‘senses’ a different molecular aspect of a given ligand structure. Indeed the same agonist exhibited functional contacts to different residues in Olfr73 than in Olfr74 resulting either in weak or strong receptor activation. Thus our findings provide molecular insight into combinatorial mechanisms of odorant signaling at the receptor level and into ligand structure-activity relationships having also impact on the design of novel activating ligands. The concept of multiple ligand-specific binding modes that induce different OR conformations and signaling responses may be a mechanism critical to the overall perception of odor.

## Methods

### Reagents

Odorants were from Firmenich SA (Geneva Switzerland), Givaudan, (Dübendorf, Switzerland) and Sigma Aldrich (Buchs, Switzerland). Polymerase chain reactions were performed using Phusion High-Fidelity DNA Polymerase (Thermo Scientific). DNA point mutations were introduced using the Quickchange Site-directed Mutagenesis Kit (Agilent Technologies). Oligonucleotide primers were from Microsynth AG (Balgach, Switzerland) and Sigma Aldrich (Buchs, Switzerland).

### Site-directed mutagenesis

The cDNA of the mouse Olfr73 (mOR-EG) was amplified from pME18SRhoEG (obtained from S. Touhara, University of Tokyo, Japan) and subcloned into the Rho-Myc pCDNA3. The Olfr74 (mOR-EV) coding sequence was amplified from mouse genomic DNA (C2C12 muscle cells genomic DNA (Bioconcept). PCRs were performed using the phusion kit and the Olfr74 gene specific primers Olfr74 (ATG ATA CTG TTC GAA AAA AAC AAT AG) and Olfr74 (TTA ATT GTG TCT ATC ATT CTT AAC AC). The Olfr73 mutant S113A, was kindly provided by K. Touhara and S. Katada (University of Tokyo, Japan). Point mutations were introduced into Olfr73 and Olfr74 cDNA using the Quickchange site-directed mutagenesis kit and the following Olfr73 and Olfr74 gene specific primers: Olfr73 (E208A) (G CTT TTC GTT TTT GCA ACG TTT AAT GC G ATT AGC ACA CTC C); Olfr73 (Y 260F) (CA ATC CTA TTC CTA TT C TGT GTA CCG AAT TCC AAG AAC TCC AG); Olfr74 (A113S) (GT GTC TTT GTA GTA ACT GAA AGC TTT TTA TTA GTG GTT ATG G); Olfr74 (A208E) (GTC TTT GCC ACA TTT AAT GAA GTC AGC ACA TTA CTC); Olfr74 (F260Y) (CC ATT TTA TTC CTT TAT TGT GTT CCC AAC TC). All mutations were verified by DNA sequencing. Mutated DNA fragments were inserted into pEAK8 vector using the HindIII/NotI restrictions sites. The triple mutants Olfr73 (S113A-E208A-Y260-F) and Olfr74 (A113S-A208E-F260Y) were obtained by consecutive PCR reactions.

### Immunocytochemistry

Hana 3A cells were seeded in an 8-well Lab-Tek II chambered coverglass with a density of 70,000 cells per well (200 μL/well) and transfected with 0.8 μg of the receptor cDNA (Olfr73, Olfr74, neurokinine 1 receptor (NK1) or a non-coding plasmid DNA control using lipofectamine. Thirty hours after transfection, cell culture chambers were placed on ice. Medium was removed and the cells were incubated for 60 min with the primary antibody solution (anti-Flag M2 IgG diluted 1:600 in staining solution (DMEM 10% containing 10 mM HEPES (4-(2-hyroxyethyl)-1-piperazine-1-ethanesulfonic acid) or anti rhodopsin 4D2 IgG diluted 1:100 in staining solution. The cells were washed with a Hank’s balanced salt solution (HBSS, Invitrogen) containing 10 mM HEPES. Then cells were incubated for 30 min in the dark with the secondary antibody solution, 1:500 anti-mouse IgG ATTO 633 from goat antiserum or 1:1000 anti-Mouse IgG-ATTO (Sigma-Aldrich), followed by two washing steps. Cells were fixed for 15 min on ice with 1% paraformaldehyde and investigated by fluorescence confocal microscoy. For immune-detection of intracellular ORs, cells were permeabilized by incubation with cold (−20 °C) methanol for 5 min. Cells were then washed 3 times with Dulbecco’s buffered saline (D-PBS, Sigma-Aldrich) and immune-detection was performed as outlined for non-permeabilized cells.

### cAMP reporter gene assay

Confluent Hana 3A cells (35000 cells per well) cultured in a 96-well plate at 37 °C (Perkin-Elmer) were transfected with 0.15 μg of wild-type or mutated Olfr73 or Olfr74 encoding plasmid DNAs and 0.15 μg of the cAMP response element secreted alkaline phosphatase reporter plasmid (pCRE-SEAP) using Lipofectamine 2000 (Invitrogen). Cells were induced 8 h after transfection with cell medium containing the ligand, or cell medium alone. For screening compound library, stock solutions of odorants (0.2–1 M) were prepared from pure compounds either in dimethyl sulfoxide (DMSO, Sigma-Aldrich, Buchs, CH) or ethanol and were stored at −20 °C. Mixtures of 5 to 10 odorants prepared form stock solutions, were diluted in serum-free DMEM and applied to the cells 8 hours after transfection (<100 μM, final individual odorant concentration). Experiments were carried out in triplicates and in parallel with a mock control (cells transfected with non-coding plasmid DNA). A relative SEAP activity [(n-1) times mock level] was calculated for each odorant mixture and those that induced an activity larger than 1 were considered for further analysis.

For odorant dose-dependent measurements of cAMP signaling specific ligands were added at increasing concentrations and measured in duplicates. Odorant-induced responses were measured using the SEAP activity reporter assay 16 h after stimulation. An aliquot of supernatant from each well was then mixed with an equal volume of 1 M diethanolamine bicarbonate (pH 9.8) containing 20 mM *p*-nitrophenyl phosphate and 1 mM MgCl_2_ (Sigma). The kinetics of hydrolysis of *p*-nitrophenol phosphate by alkaline phosphatase was determined by measuring the absorbance at 410 nm using a multiwell plate reader (SpectraMax 360, Molecular Devices). To remove SEAP activity background noise from the measurements, the mean value of mock control SEAP activity (Supplementary Figures 8 and 9) was subtracted from the mean value of OR-induced SEAP activity for each odorant concentration. Dose response curves and EC_50_ values were then determined by fitting these points with the Hill equation using IGOR Pro software (WaveMetrics, Portland, OR, USA). Statistical significance of the differences in EC_50_ values was analyzed using a two-tailed Student’s t test. p values below 0.05 were considered as being significant.

### Homology modeling of Olfr73 and Olfr74

The initial homology models of Olfr73 and Olfr74 were obtained using Modeller 9.10 41 based the on crystal structure of the β2-adrenergic receptor^42^ (pdb code: 3ny9). The β_2_-adrenergic receptor shares 16.4% and 16.8% sequence identity with Olfr73 and Olfr74, respectively. The sequence alignments were performed automatically in ClusterW2^43^ and adjusted manually in Discovery Studio Visualizer for proper aligning of conserved motifs and disulfide bridges. 1500 initial models (500 × 3 with different random seeds) were generated for both Olfr73 and Olfr74 using Modeller with fully annealed protocol; the optimal model was then chosen for subsequent studies according to DOPE (Discrete Optimized Protein Energy) score.

### Model refinement in Prime

The initial Olfr73 and Olfr74 models generated with Modeller were aligned with the β_2_-adrenergic receptor crystal structure (pdb code: 3ny9) from OPM (Orientations of Proteins in Membranes) database^44^. The pre-aligned Olfr73 and Olfr74 models were imported into the Maestro9.2 program. Hydrogens were added to the structure according to physiological pH. The protein preparation utility in Maestro9.2 was used to run a restrained minimization which removed unfavorable steric contacts and improved the quality of the protein hydrogen bonding network without large rearrangements of the protein heavy atoms. Pre-prepared models were submitted to Prime in Schroedinger2011 for constrained truncated-Newton minimization refinement, using the OPLS_2005 force field and implicit membrane model.

### Full atomic model refinement in Desmond

Restrained molecular dynamics simulations were performed to achieve local improvements of the homology models. Using the system builder of Desmond^45^ in the Maestro 9.2 program, well prepared Olfr73 and Olfr74 models were embedded into a pre-equilibrated palmitoyl-oleolyl-glycerophosphatidylethanolamine (POPE) lipid bilayer solvated in 0.15 M NaCl. All protein residues were modeled according to their protonation state at neutral pH, leaving the protein with a net charge of +7e. The total number of atoms was about 43,600 including 23 Na^+^ and 30 Cl^−^ ions, about 8,200 water molecules, and 125 POPE molecules. The system covered a volume of 70 × 61 × 100 Å^3^. CHARMM36 force field was assigned to the membrane system by VIPARR in Desmond. The whole system was heated linearly at constant volume (NVT ensemble, 1bar) from 0 to 310 K over 400 ps. Equilibration was obtained using the Desmond protocol at constant pressure and temperature (NPT ensemble; 310 K, 1 bar) and the Berendsen coupling scheme with one temperature group. All bond lengths of hydrogen atoms were constrained using M-SHAKE. Van der Waals and short-range electrostatic interactions were cut off at 10 Å. Long-range electrostatic interactions were computed using particle mesh Ewald (PME) summation. A time-reversible reference system propagator algorithm (RESPA) was used for 1.0 fs time step integration; long-range electrostatics were computed every 4.8 fs. In each simulation, harmonic positional restraints on the protein backbone were tapered off linearly from 10 to 0.3 kcal mol^−1^ Å^−2^ over 30 ns. Additional 10 ns equilibrations restrained the protein alpha-carbons with 0.3 kcal mol^−1^ Å^−2^ force constant. Finally, 50 ns MD simulations with 2.0 fs time steps were performed for both Olfr73 and Olfr74 constraining alpha carbon at 0.3 kcal mol^−1^ Å^−2^ force constant.

### Remodeling Olfr73

For a better comparison with Olfr74, new Olfr73 models were regenerated in Modeller 9.10 using the above-mentioned protocol.

### Ligand preparation and docking

All ligand structures were obtained from PubChem online database (http://pubchem.ncbi.nlm.nih.gov/). Ligand preparation utility was used to optimize the geometry of initial structures. Systematic conformation searches in MacroModel^46^ yielded refined models of which the lowest potential energy poses were kept for docking.

The docking was performed with Glide^47^ distributed in Schrodinger2011 suite. Ligand molecules were initially placed at the binding pocket near ECL2 region with random poses. The docking region was defined by a 10 Å radius surrounding the ligand mass center. Glide docking and scoring was performed for all cases. Twenty poses per ligand were included in post-docking minimization for optimizing bond lengths and angles as well as torsional angles. The best-scored poses were used for analyses.

## Additional Information

**How to cite this article**: Baud, O. *et al.* Exchanging ligand-binding specificity between a pair of mouse olfactory receptor paralogs reveals odorant recognition principles. *Sci. Rep.* **5**, 14948; doi: 10.1038/srep14948 (2015).

## Supplementary Material

Supplementary Information

## Figures and Tables

**Table 1:** Activation of Olfr73 wildtype and mutants by specific odorants.

Olfr73					
EC_50_(μM)	wt	S113A	E208A	Y260F	S113A-E208A-Y260F
Jasmone	—	—	66 ± 9	—	83 ± 11
Rose Oxide	35 ± 20	23 ± 24	10 ± 3	—	—
Nootkatone	225 ± 76	—	277 ± 54	—	—
Metyl vanillate	241 ± 42	—	347 ± 32	—	—
MIEG	131 ± 19	—	10 ± 5	—	—
Ethyl vanillin	35 ± 4	202 ± 44	147 ± 11	246 ± 20	—

EC_50_ values (μM) of jasmone, rose oxide, nootkatone, methyl vanillate, methyl isoeugenol (MIEG), and ethyl vanillin obtained from dose-response curves using the SEAP reporter assay measurement on wild-type and mutant receptors of Olfr73 as shown in Supplementary Fig. 4. Experiments were performed in triplicate and data are given as means ± standard deviation. Amino acids are indicated in one-letter code.
