# Supplementary material for: Exchanging ligand-binding specificity between a pair of mouse olfactory receptor paralogs reveals odorant recognition principles
Source: Sci Rep. 2015 Oct 9;5:14948. doi: 10.1038/srep14948 (PMC4598832; doi:10.1038/srep14948)
Supplement: Supplementary Information [file srep14948-s1.pdf]

Supplementary information for the article

Exchanging ligand-binding specificity between a pair of mouse olfactory receptor paralogs reveals odorant recognition principles

Olivia Baud *, Shuguang Yuan *, Luc Veya, Slawomir Filipek, Horst Vogel, Horst Pick

* contributed equally

Correspondence: H.V. (horst.vogel@epfl.ch), H.P. (horst.pick@epfl.ch)

Content:

Supplementary Figures……………………………………………………..p 2–10

Supplementary Table…………………………………………………….p 11

Supplementary List of Odorant Compounds……………………………….p 12-32

Supplementary Figures

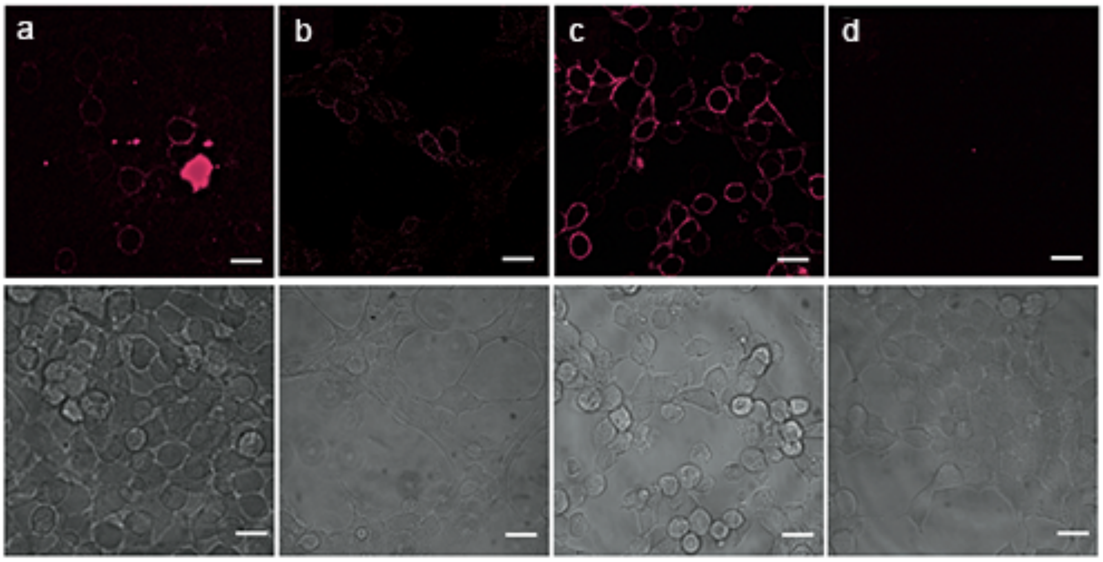

Supplementary Figure 1: **Functional expression of odorant receptors at the cell membrane of Hana3A cells**. Immuno-detection of (**a**) Olfr74, (**b**) Olfr73, (**c**) the neurokinin receptor-1 (NK1) a highly expressed prototypic GPCR, and (**d**) not transfected negative control cells. Confocal images show the cell surface localization of the receptors by antibody detection (upper panel), and the corresponding transmission images (lower panel). Immunolabeling was performed using a monoclonal anti-Flag antibody and a goat anti-mouse IgG conjugated with ATTO 633 (Excitation: 633 nm/emission filter: LP 650 nm). Scale bars: 20 µm.

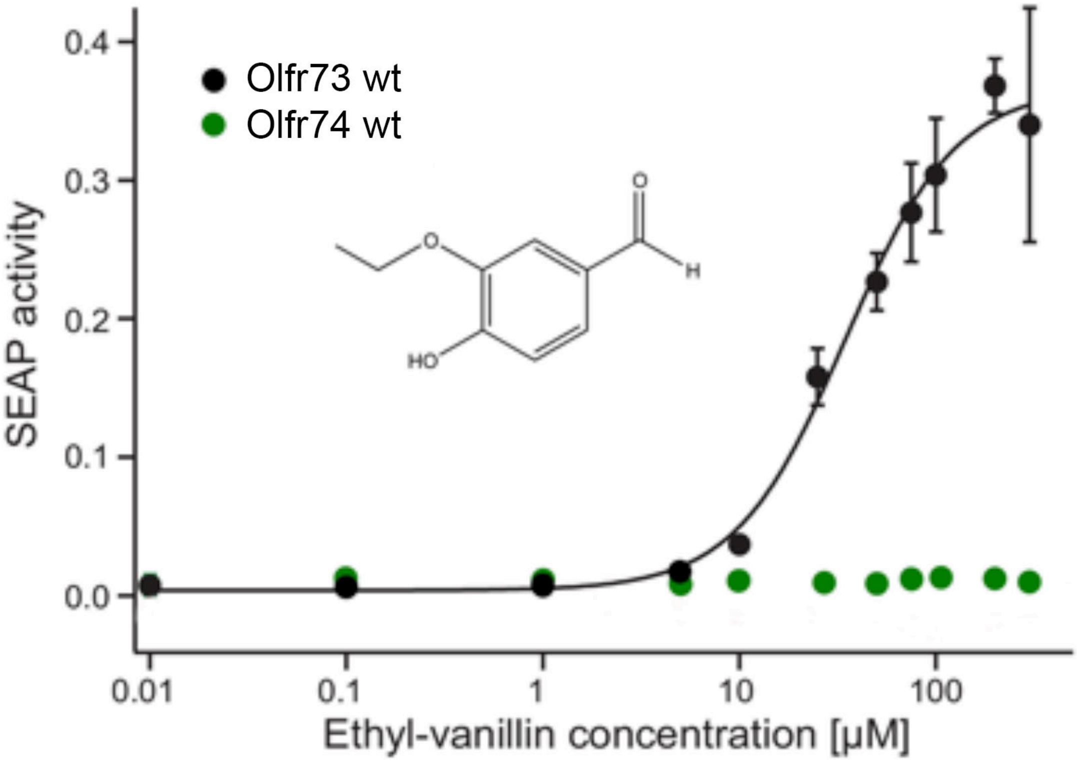

Supplementary Figure 2. **Concentration-dependent OR activation by ethyl-vanillin**. Secreted alkaline phosphatase (SEAP) reporter activity of Olfr73 (black filled circles), and Olfr74 (green filled circles) after stimulation with increasing concentrations of EV (inset: chemical structure EV). Reporter activity of the mock-transfected cells was subtracted from the OR activity values. For activation of Olfr73 by ethyl vanillin an EC_{50} value of 35 ± 4 μM was obtained. Olfr74 showed no detectable activation by ethyl vanillin. Experiments were performed in triplicates.

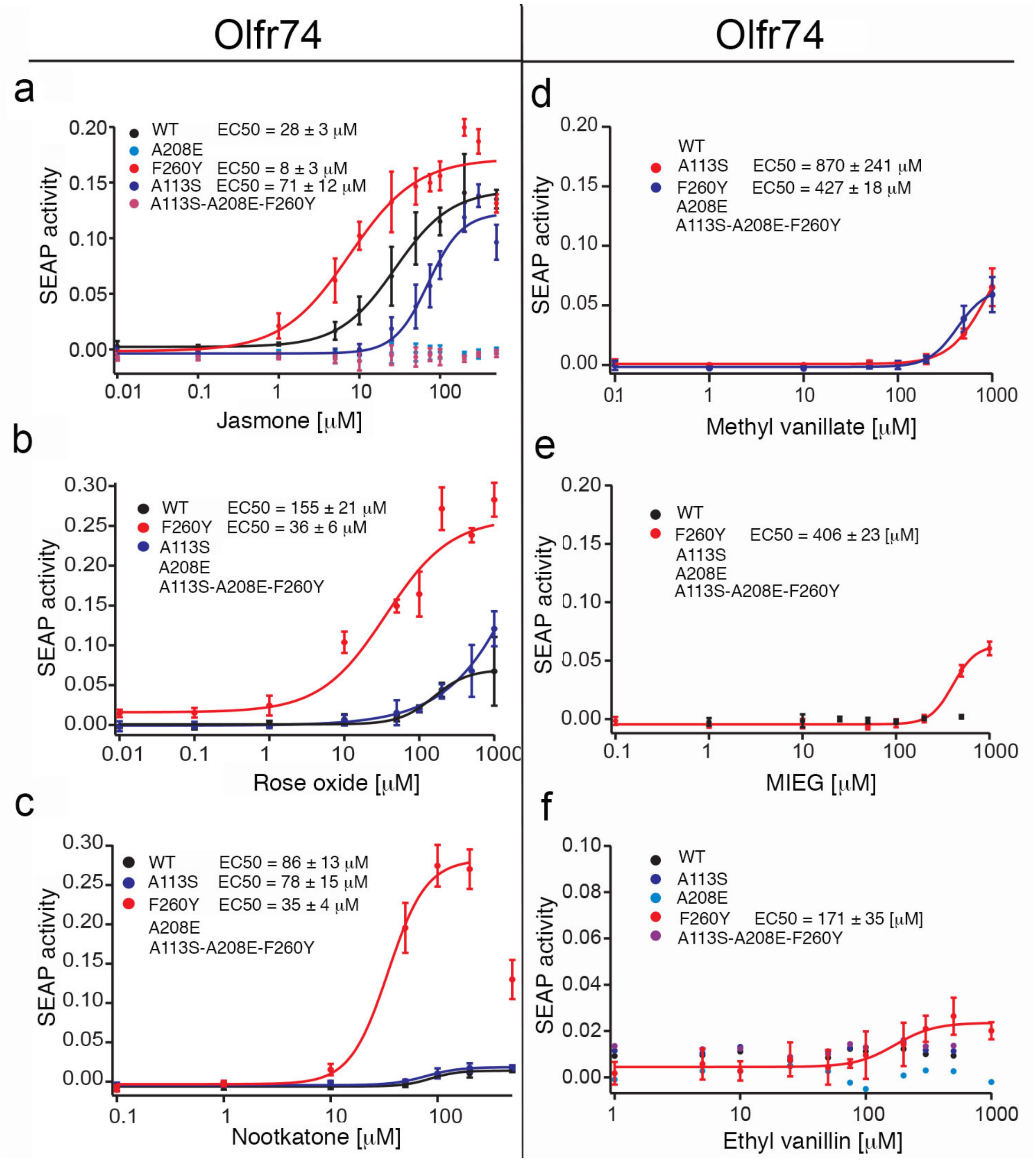

Supplementary Figure 3: **Activation of wildtype and mutant Olfr74 by specific odorants**. Dose response curves of (**a**) jasmone (**b**) rose oxide, (**c**) nootkatone, (**d**) methyl vanillate, (**e**) methyl isoeugenol (MIEG), and (**f**) ethyl vanillin measured on Olfr74 wildtype (WT) activation and specific point mutants of its putative ligand binding site, that increase sequence homology with Olfr73. Secreted alkaline phosphate reporter activity (SEAP) was determined in response to Olfr74 wt or Olfr74 mutant receptor activation by increasing concentrations of the respective ligands. EC_{50} values are indicated for those cases where odorant responses were measurable.

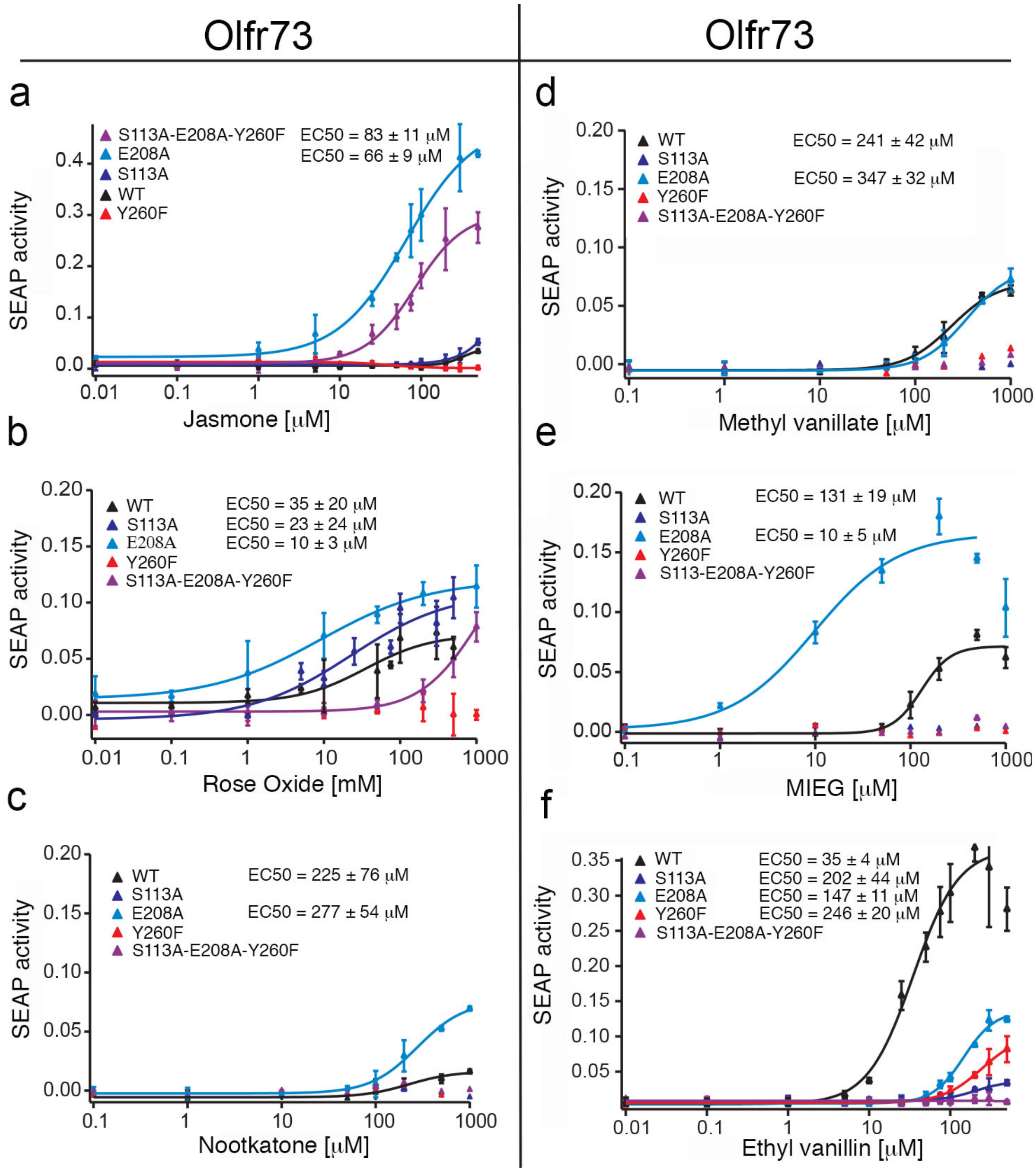

Supplementary Figure 4: **Activation of wildtype and mutant Olfr73 by specific odorants**. Dose response curves of (**a**) jasmone (**b**) rose oxide, (**c**) nootkatone, (**d**) methyl vanillate, (**e**) methyl isoeugenol (MIEG), and (**f**) ethyl vanillin measured on Olfr73 wildtype (WT) and specific point mutants of its putative ligand binding site, that increase sequence homology with Olfr74. Secreted alkaline phosphate reporter activity (SEAP) was determined in response to Olfr73 wt or Olf73 mutant receptor activation by increasing concentrations of the respective ligands. EC_{50} values are indicated for those cases where odorant responses were measurable.

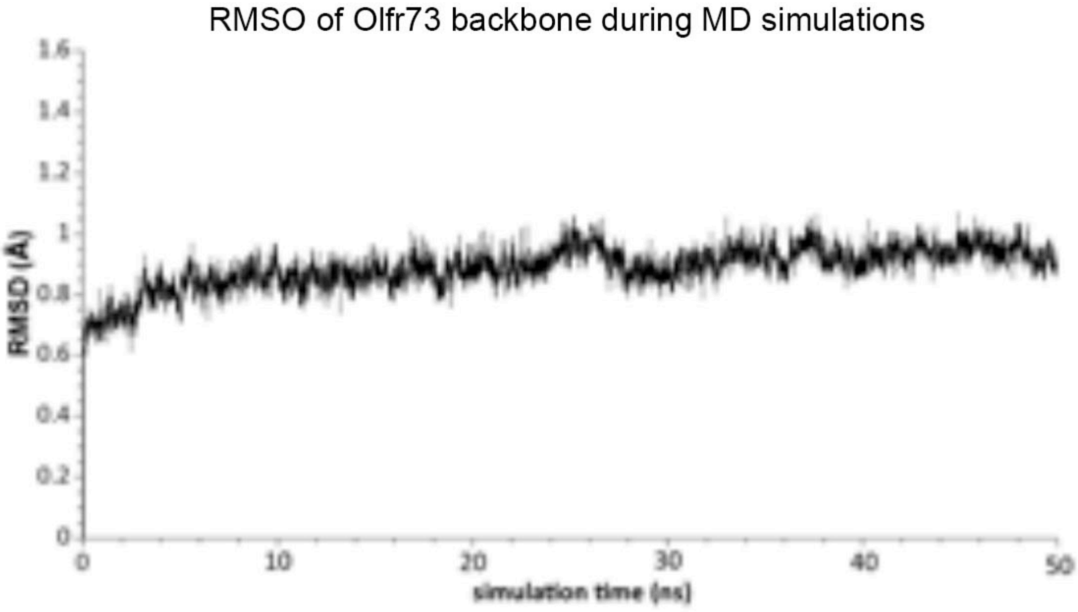

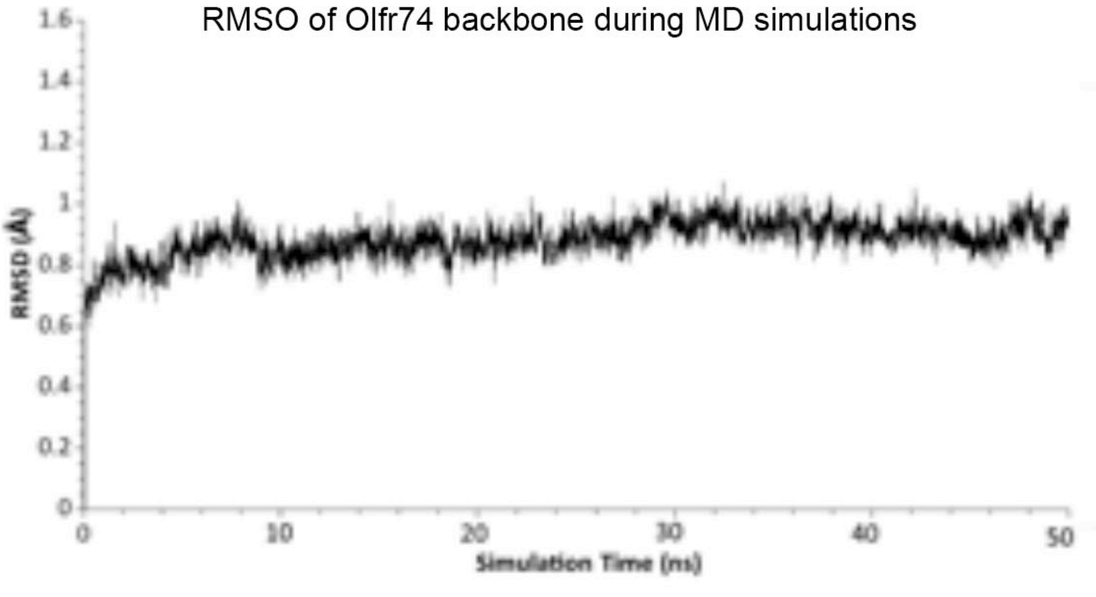

Supplementary Figure 5: **RMSD of Olfr73 (top panel) and Olfr74 (bottom panel) backbone during MD simulations**. No significant movements were found during the whole 50 ns MD productions for both cases.

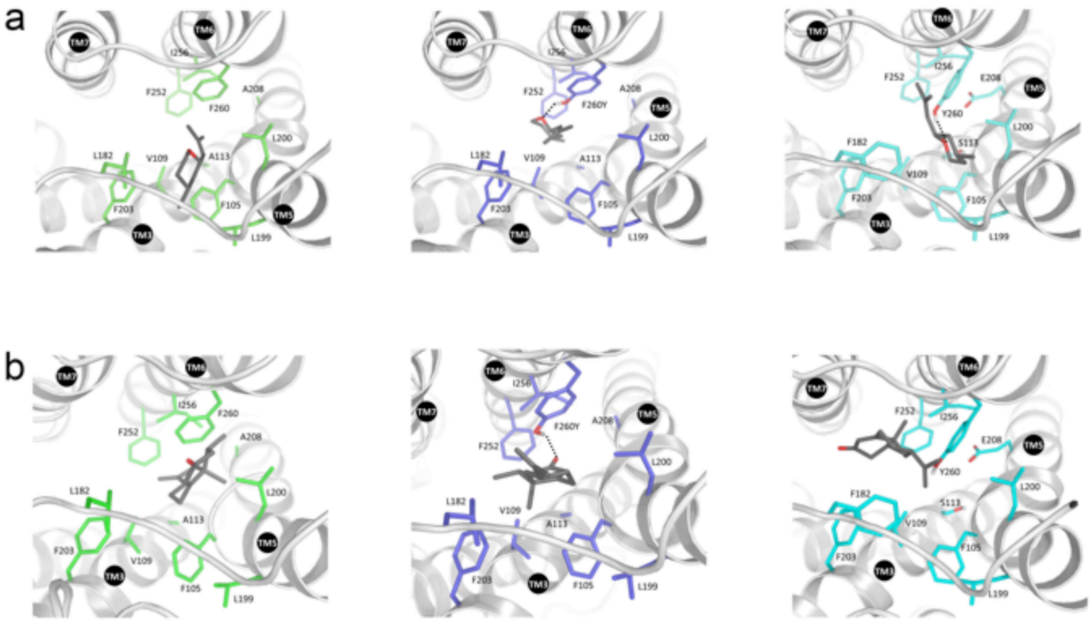

Supplementary Figure 6: **Top view of rose oxide and nootkatone docked in the calculated Olfr73 and Olfr74 models.** (**a**) left: rose oxide docked in wildtype Olfr74; middle: rose oxide docked in Olfr74 (F260Y); right: rose oxide docked in wildtype Olfr73 (**b**) left: nootkatone docked in wildtype Olfr74; middle: nootkatone docked in Olfr74(F260Y); right: nootkatone docked in wildtype Olfr73.

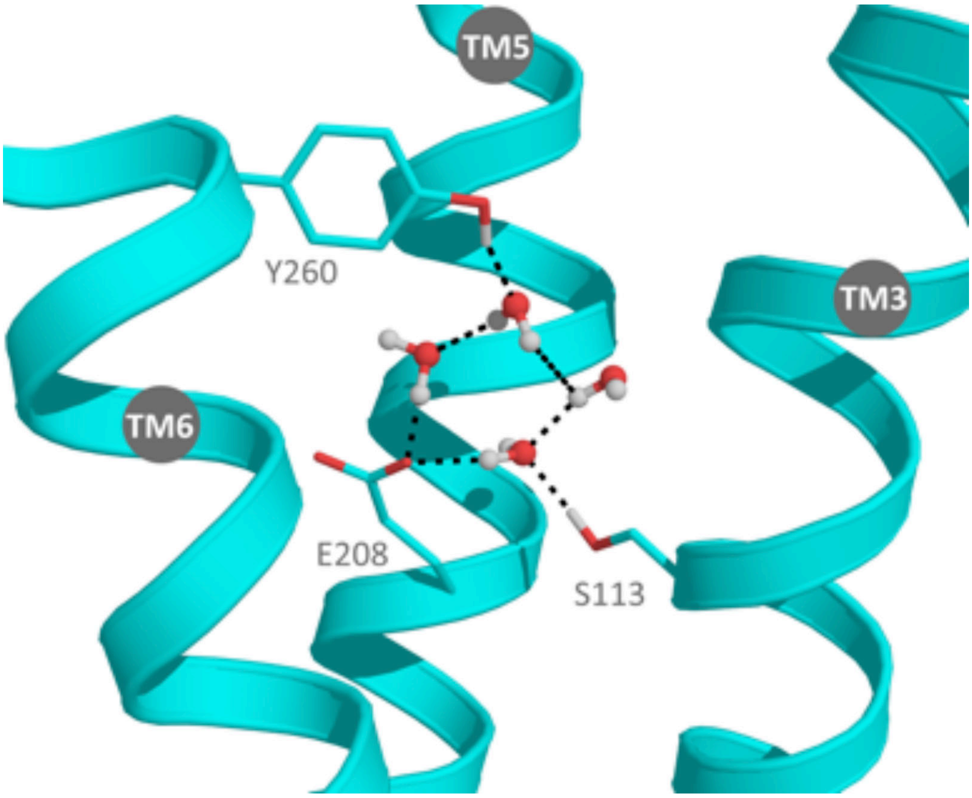

Supplementary Figure 7: **Solvent mediated hydrogen bond between Y260, E208 and S113 in Olfr73 MD simulations**.

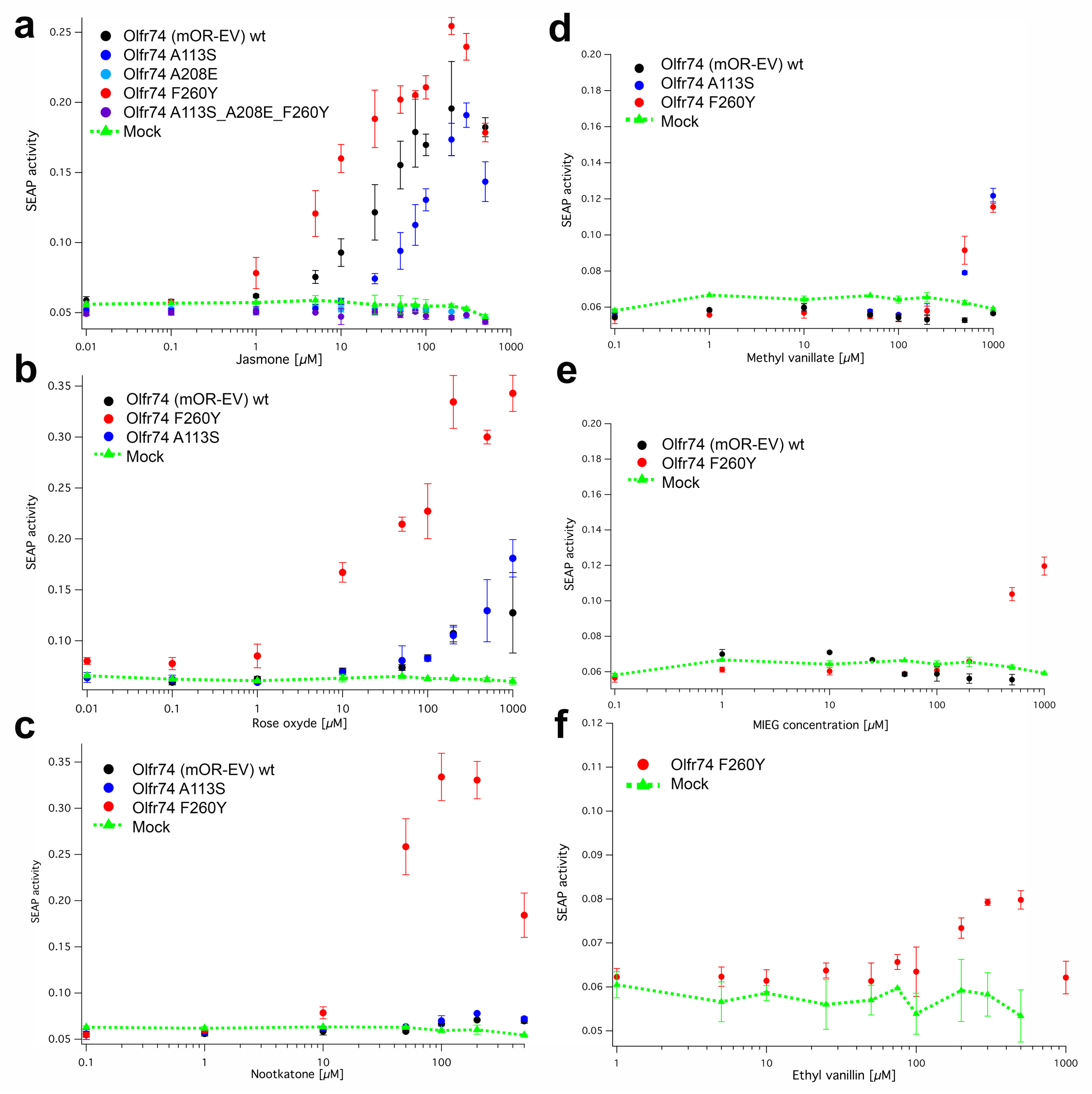

Supplementary Figure 8: **Mock control compared to activation of wildtype and mutant Olfr74 by specific odorants**. Different odorants do not show significant activation of non-transfected cells (Mock control: green dotted line). Activation of mock control, Olfr74 wt and different Olfr74 point mutants by (**a**) jasmone (**b**) rose oxide, (**c**) nootkatone, (**d**) methyl vanillate, (**e**) methyl isoeugenol (MIEG), and (**f**) ethyl vanillin. Secreted alkaline phosphate reporter activity (SEAP) was determined in response to increasing concentrations of the respective ligands. Graphs represent the raw data of dose-response curves shown in Supplementary Figure 3.

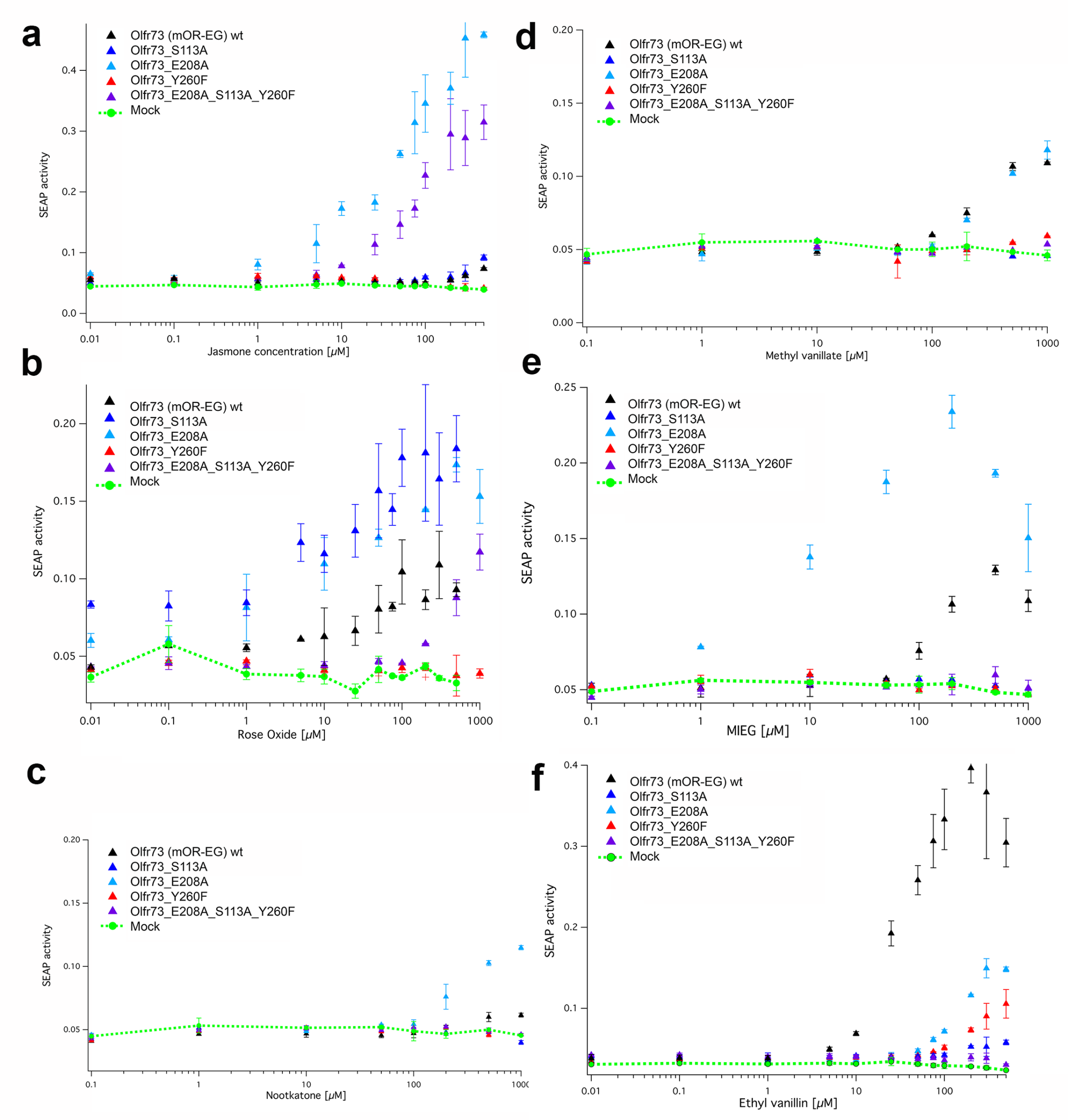

Supplementary Figure 9: **Mock control compared to activation of wildtype and mutant Olfr73 by specific odorants**. Different odorants do not show significant activation of non-transfected cells (Mock control: green dotted line). Activation of mock control, Olfr73 wt and different point mutants of Olfr73 by (**a**) jasmone (**b**) rose oxide, (**c**) nootkatone, (**d**) methyl vanillate, (**e**) methyl isoeugenol (MIEG), and (**f**) ethyl vanillin. Secreted alkaline phosphate reporter activity (SEAP) was determined in response to increasing concentrations of the respective ligands. Graphs represent the raw data of dose-response curves shown in Supplementary Figure 4.

Supplementary Table

MMGBSA Binding Affinity Evaluation for Docking				
**Jasmone**				
	**Olfr73 (WT)**	**Olfr73 (E208A)**	**Olfr74 (WT)**	**Olfr74 (F260Y)**
**EC50**	>100	70	30	10
**Binding Energy (kcal/mol)**	-42.1	-46.8	-53.5	-55.8
**Restrained Energy (kcal/mol)**	3.3	0.7	0.7	0.8
**Nootkatone**				
	**Olfr73 (WT)**	**Olfr73 (E208A)**	**Olfr74 (WT)**	**Olfr74 (F260Y)**
**EC50**	>100	>100	90	40
**Binding Energy (kcal/mol)**	-55.7	-58.5	-56.7	-61.9
**Restrained Energy (kcal/mol)**	4.4	3.0	0.4	0.5
**Rose Oxide**				
	**Olfr73 (WT)**	**Olfr73 (E208A)**	**Olfr74 (WT)**	**Olfr74 (F260Y)**
**EC50**	30	20	>100	40
**Binding Energy (kcal/mol)**	-48.8	-51.5	-42.7	-44.3
**Restrained Energy (kcal/mol)**	0.7	0.6	0.2	0.2

Supplementary Table 1: Odorant binding affinity calculations performed in Prime based on Glide docking results. Residues around 4 Å distance of the docked ligand were treated to be flexible during the evaluation. Both ligand binding energy and ligand restrain energies were calculated. EC_{50} values were derived from Fig. 2 (main manuscript), and Supplementary Fig. 4.

Supplementary list of odorant compounds

A	1	Lyral	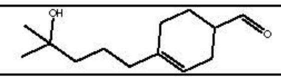
A	2	Lilial	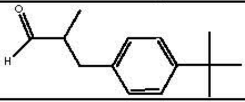
A	3	Geranyl formate	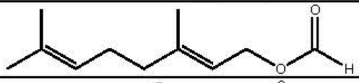
A	4	Cyclosal	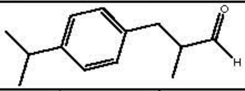
A	5	Farenal HR	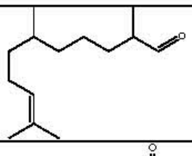
A	6	Geranyl acetate extra	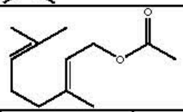
B	7	Farnesol	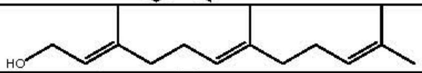
B	8	Foliaver	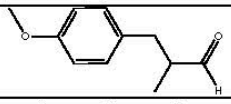
B	9	Geraniol	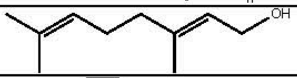
B	10	Phenethyl alcohol	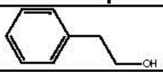
B	11	Heliopropanal	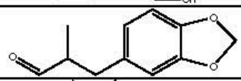
B	12	Citronellal CP	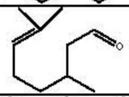
C	13	Methalcinnamaldehyde	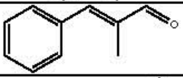
C	14	Phenylethyl acetate	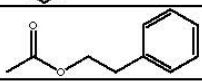
C	15	Decenal	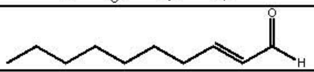
C	16	Phenethyl formate	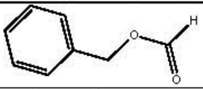
C	17	Herbaldehyde	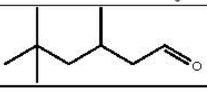

**D**	**18**	Heliotropine	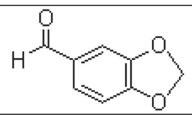
**D**	**19**	Guaiacol	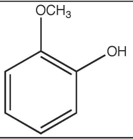
**D**	**20**	Cinnamyl alcohol ord	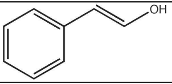
**D**	**21**	Pentanol-1	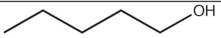
**D**	**22**	Tarragol	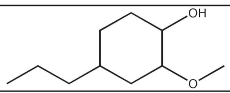
**D**	**23**	Octahydrocoumarine	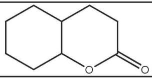
**E**	**24**	Capric acid (decanoic acid)	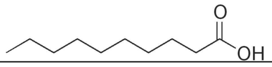
**E**	**25**	Caprylic acid (octonoic acid)	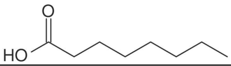
**E**	**26**	Heptanoic acid	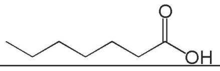
**E**	**27**	Caproic acid (hexanoic acid)	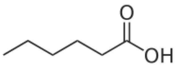
**E**	**28**	Valeric acid (pentanoic acid)	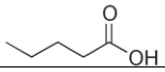
**E**	**29**	Methyl butyrate	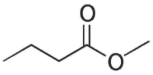
**E**	**30**	Methyl valerate	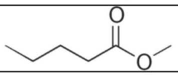
**F**	**31**	Methyl caproate	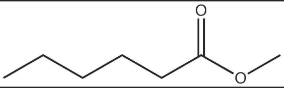
**F**	**32**	Methyl heptanoate	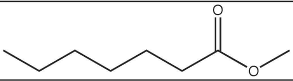
**F**	**33**	Methyl caprylate	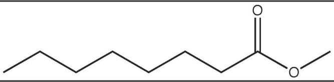
**F**	**34**	Methyl nonanoate	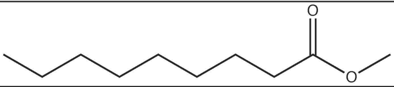

**F**	**35**	Ethyl butyrate	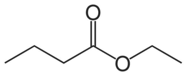
**F**	**36**	Ethyl valerate	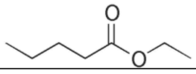
**F**	**37**	Ethyl caproate	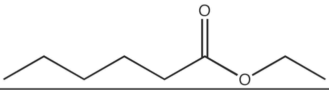
**F**	**38**	Pentyl valerate	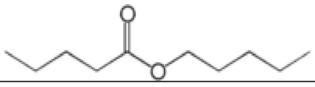
**G**	**39**	Pentanone-2	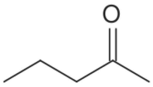
**G**	**40**	Hexanone-2	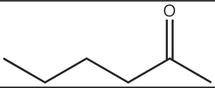
**G**	**41**	Heptanone-2	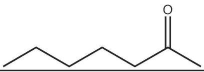
**G**	**42**	Octanone-2	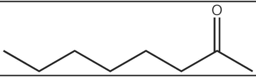
**G**	**43**	Nonanone-2	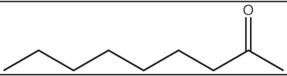
**G**	**44**	Pentanone-3	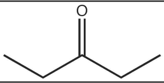
**G**	**45**	Hexanone-3	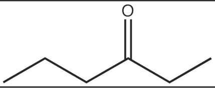
**G**	**46**	Octanone-3	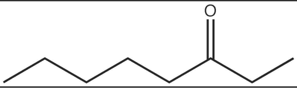
**H**	**47**	Butanol-1	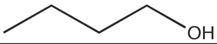
**H**	**48**	Pentanol-1	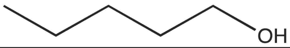
**H**	**49**	Hexanol-1	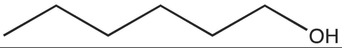
**H**	**50**	Heptanol-1	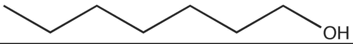
**H**	**51**	Octanol-1	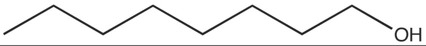

**H**	**52**	Butanol-2	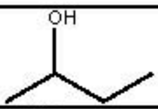
**H**	**53**	Pentanol-2	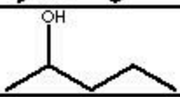
**H**	**54**	Hexanol-2	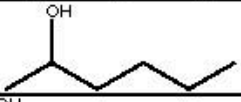
**H**	**55**	Octanol-2	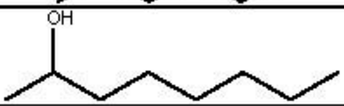
**I**	**56**	Pipol	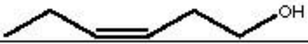
**I**	**57**	Carbinol PV (3-octen-1-ol)	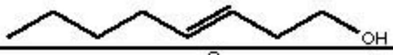
**I**	**58**	Isobutyraldéhyde	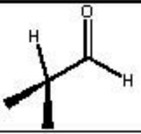
**I**	**59**	Isovaléraldéhyde	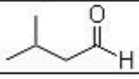
**I**	**60**	Hexenal-2	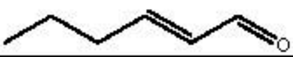
**I**	**61**	Heptanal	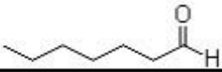
**I**	**62**	Octanal	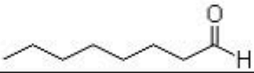
**I**	**63**	Decenal-2	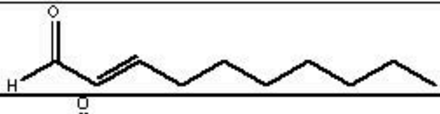
**I**	**64**	Octenal-2	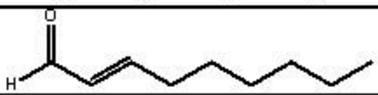
**I**	**65**	Melonal	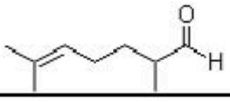
**J**	**66**	Citral	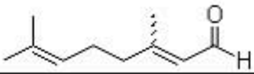
**J**	**67**	Hydroxy citronellal	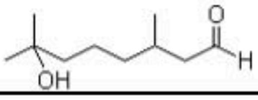

J	68	Tangerinal	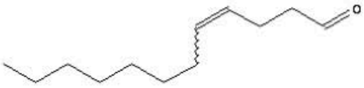
J	69	Dodecanal	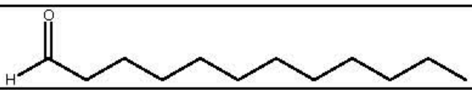
J	70	Aldehyde MNA	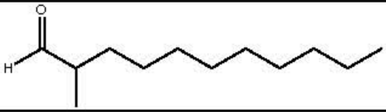
J	71	Hexanenitril	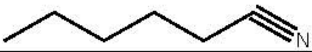
J	72	Octanenitril	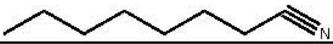
J	73	Decanenitril	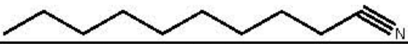
J	74	Geranyl nitril	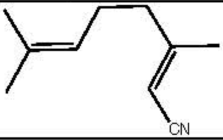
J	75	2-Methyldecanenitril	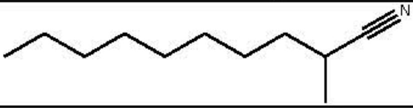
K	76	Jasmonitril	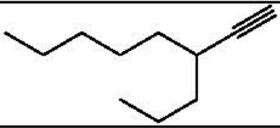
K	77	Myrcene	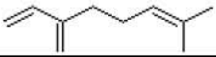
K	78	Undecantriene	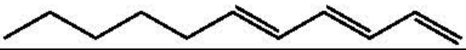
K	79	Linalool	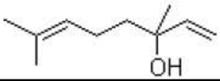
K	80	Linalyl acetate	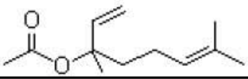
K	81	Nerolidol	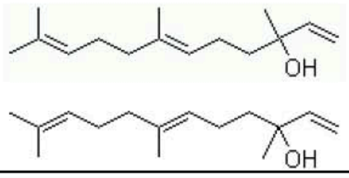
K	82		-

**K**	**83**	Dibutylsulfure	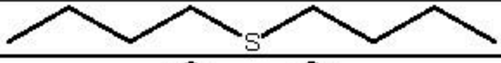
**K**	**84**	Diethylamine	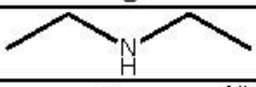
**K**	**85**	Butylamine	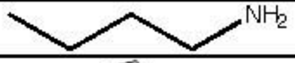
**K**	**86**	Cyclopentanone	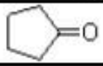
**K**	**87**	Pentylendo	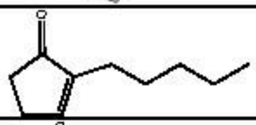
**K**	**88**	Veloutone	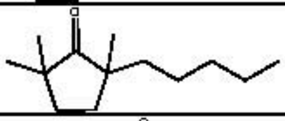
**L**	**89**	Cyclohexanone	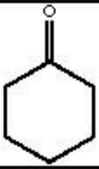
**L**	**90**	Methyl-2-Cyclohexanone-1	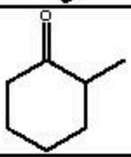
**L**	**91**	Dimethyl-4-4-Cyclohexanone-1	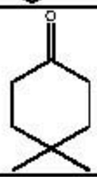
**L**	**92**	Dihydroisophorone	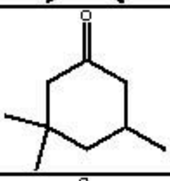
**L**	**93**	Tetramethyl-2,2,6,6-cyclohexanone-1	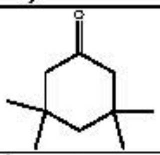
**L**	**94**	Tert-Butyl-4-Cyclohexanone	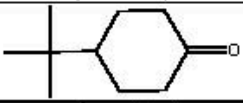
**L**	**95**	Tert-Butyl-2-Cyclohexanone	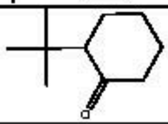

M	96	Norbornanone	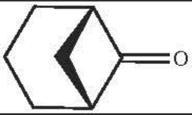
M	97	Fenchone (+/-)	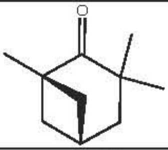
M	98	Nopinone	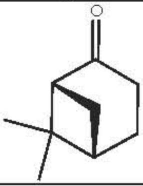
M	99	Cyclooctanone	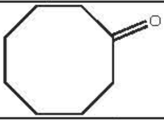
M	100	Cyclodécanone	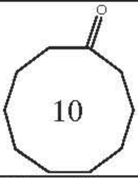
M	101	Cyclododecanone	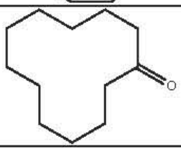
M	102	Muscone	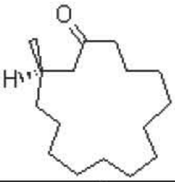
M	103	Civetone	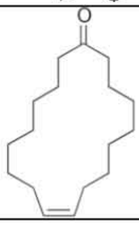
N	104	Menthone (+/_)	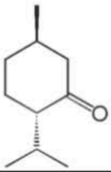

N	105-106	Carvone (+/-)	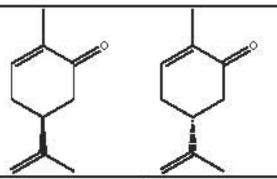
N	107	Camphor (+/-)	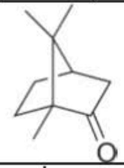
N	108	Z-95	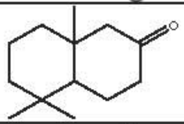
N	109	Tamisone	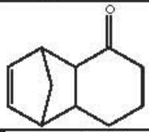
N	110	Orivone	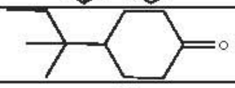
N	111	4-tert-butyl-1-cyclohexanol	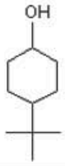
N	112	Lorisia / Dorisyl	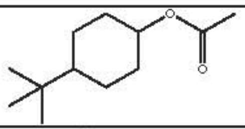
N	113	Beta-ionone	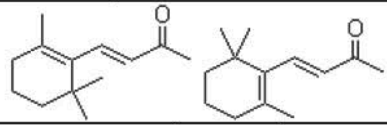
O	114	Alfa-ionone	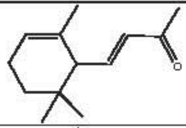
O	115	Beta-damascone	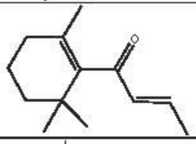
O	116	Alfa-damascone	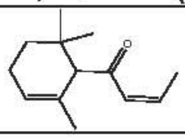

**O**	**117**	Beta-damascenone	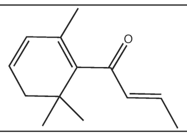
**O**	**118**	Toluene	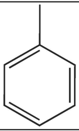
**O**	**119**	P-Cymene	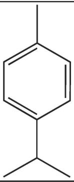
**O**	**120**	O-xylene	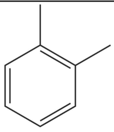
**O**	**121**	M-xylene	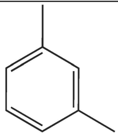
**O**	**122**	P-xylene	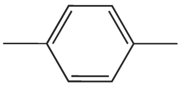
**O**	**123**	Phenol	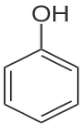
**P**	**124**	O-cresol	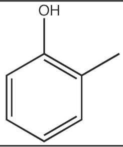
**P**	**125**	M-cresol	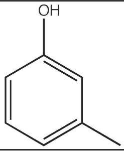
**P**	**126**	P-cresol	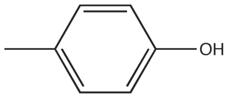

P	127	2-methylphenyl acetate	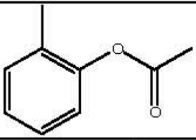
P	128	3-methyl-1-phenyl acetate	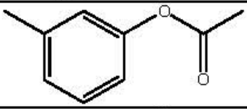
P	129	4-methylphenyl acetate	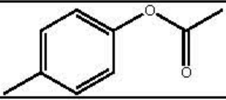
P	130	2-tert-butylphenol	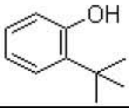
P	131	3-tert-butylphenol	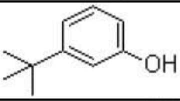
P	132	4-tert-butylphenol	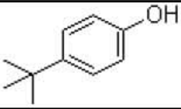
P	133	Anisole	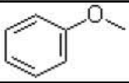
Q	134	Estragole	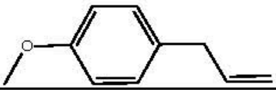
Q	135	Anethole	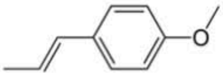
Q	136	-	
Q	137	-	
Q	138	Aniline	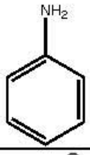
Q	139	Benzaldehyde	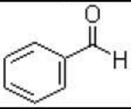
Q	140	Acetophenone	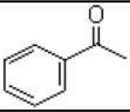

**Q**	**141**	Aldehyde salicylique	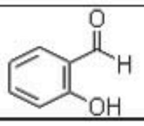
**Q**	**142**	Methyl salicylate	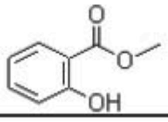
**Q**	**143**	Isobutyl salicylate	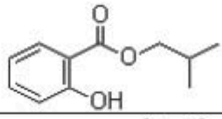
**Q**	**144**	Cyclohexyl salicylate	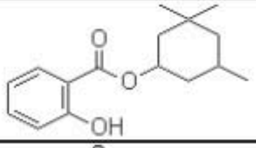
**Q**	**145**	Benzyl salicylate	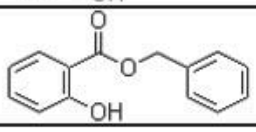
**Q**	**146**	Methyl anthranylate	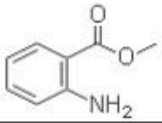
**R**	**147**	Methyl N-methylanthranilate	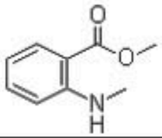
**R**	**148**	Methyl phenylacetate	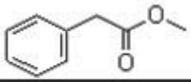
**R**	**149**	Benzyl alcohol	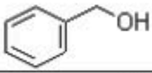
**R**	**150**	Acetate de benzyl	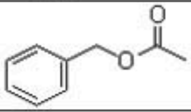
**R**	**151**	2-phenyl-1-ethanol	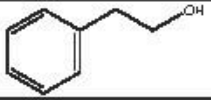
**R**	**152**	2-phenylethyl acetate	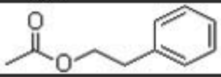

**R**	**153**	Alcohol cinnamique	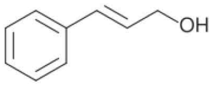
**R**	**154**	Cinnamyle acetate	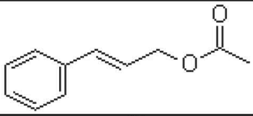
**R**	**155**	Methyl benzoate	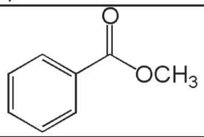
**R**	**156**	Methyl vanillate	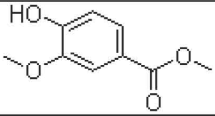
**S**	**157**	Helional	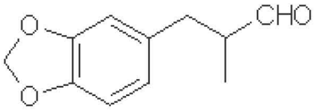
**S**	**158**	Methyl anisate	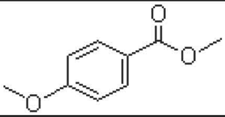
**S**	**159**	Coumarine	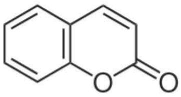
**S**	**160**	Vanillin	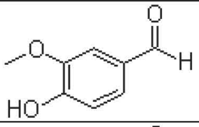
**S**	**161**	Isovanillin	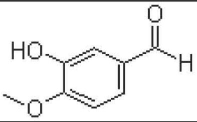
**S**	**162**	4-hydroxy-3-methoxybenzyl-alcohol	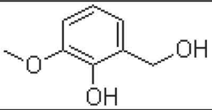
**S**	**163**	Isoeugenol	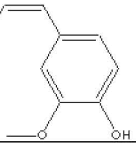
**S**	**164**	Eugenol	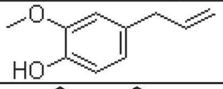
**S**	**165**	Safrole	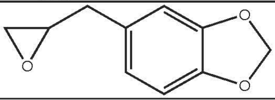

T	166	3,4,Methylenedioxyacetophenone	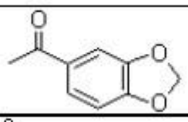
T	167	Piperonyl acetate	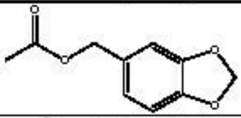
T	168	Piperonyl alcohol	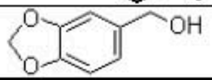
T	169	4-isopropylphenol	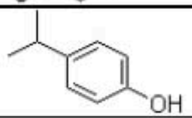
T	170	-	
T	171	Veratryl aldehyde	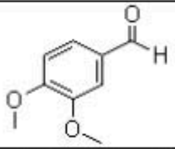
T	172	4-methyl veratrole	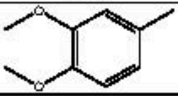
T	173	Aldehyde TPM	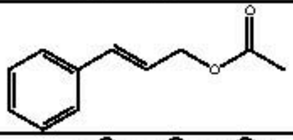
T	174	Foliaver	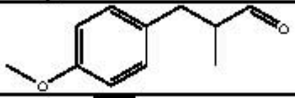
T	175	Raspberry ketone	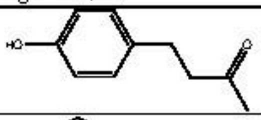
T	176	Calone	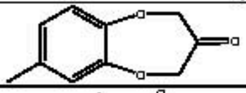
U	177	Mousse crystal	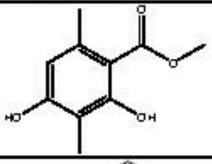
U	178	Ambrox	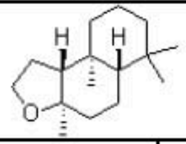
U	179	Oxy-caryophyllene	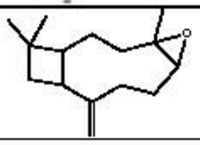

**U**	**180**	Patchoulol	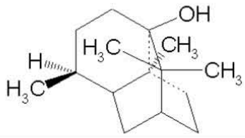
**U**	**181-182**	(-/+)-limonene	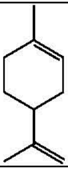
**U**	**183**	Alpha-pinene	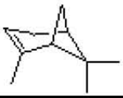
**U**	**184**	Cyclomethylene citronellol	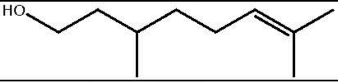
**U**	**185**	Liminal	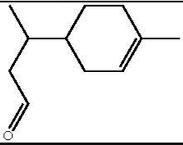
**U**	**186**	Morpholin	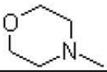
**U**	**187**	-	
	**188**	Piperidine	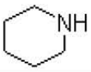
	**189**	Maltol	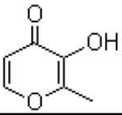
	**190**	Pyridine	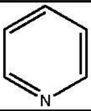
	**191**	Sotolone	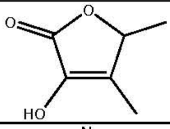
**V**	**192**	Pyrazine	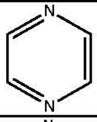
**V**	**193**	Trimehylpyrazine	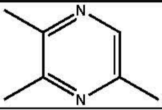

**V**	**194**	Menthenethiol-p	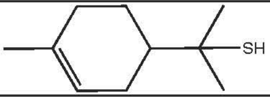
**V**	**195**	Geosmin	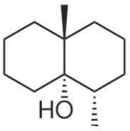
**V**	**196**	Eucalyptol	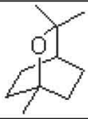
**V**	**197**	Oxane	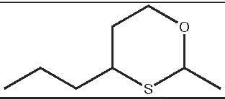
**V**	**198**	Rose oxide	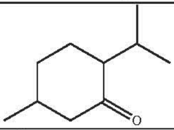
**V**	**199**	Dynascone	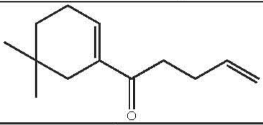
**V**	**200**	Z95	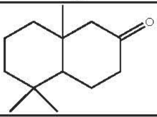
**V**	**201**	Polywood super	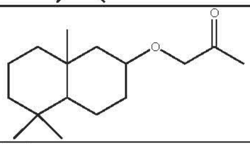
**W**	**202**	Boisambrene forte	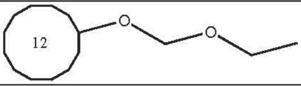
**W**	**203**	Palisandin	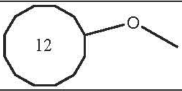
**W**	**204**	Indol	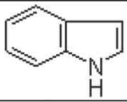
**W**	**205**	Isobutylquinoline	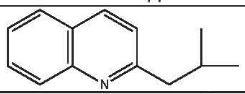
**W**	**206**	Anisyl acetate	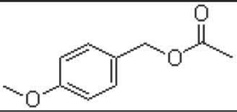
**W**	**207**	Aldehyde anisique	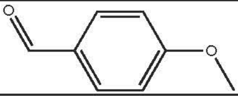

W	208	Nitrobenzene	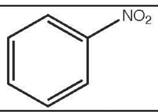
W	209	Naphtalene	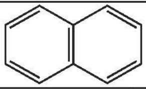
W	210	Methylnaphthyl cetone	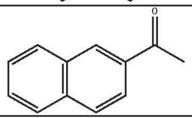
W	211	Diphenyloxide	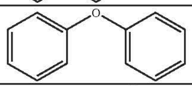
	212	Amylcinnamic aldehyde	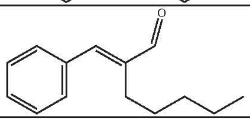
	213	Cinnamyl cinnamate	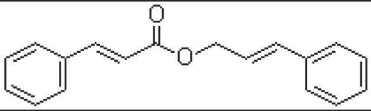
	214	Jasmone	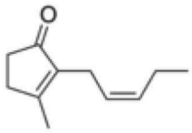
X	215	Hedione	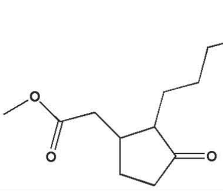
X	216	Aldehyde paratoluique	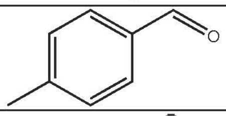
X	217	Cuminaldehyde	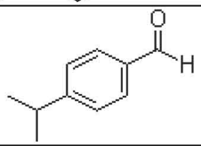
X	218	Perillaldehyde	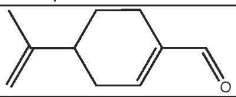
X	219	-	
X	220	-	
X	221	Cashmeran	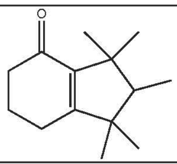

**X**	**222**	Herbanate	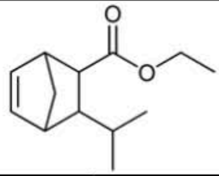
**X**	**223**	Vertonal	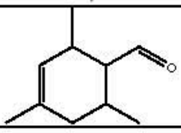
**X**	**224**	Isopropyl acetate	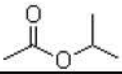
**Y**	**225**	Butyl acetate	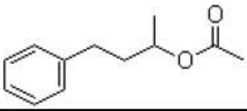
**Y**	**226**	Isoamyl acetate	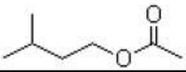
**Y**	**227**	-	
**Y**	**228**	Pipol acetate	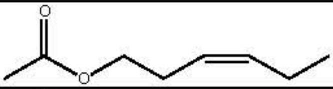
**Y**	**229**	Octyl acetate	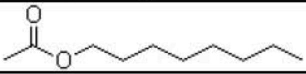
**Y**	**230**	Decyl actate	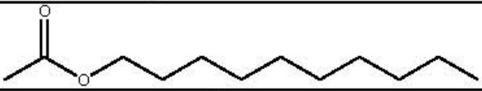
**Y**	**231**	5-nonanyl acetate	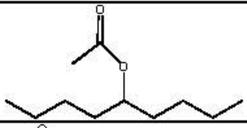
**Y**	**232**	Carbinol acetate	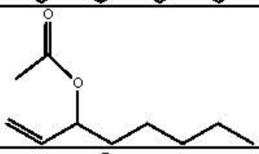
**Z**	**233**	Cyclopentyl acetate	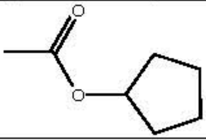
**Z**	**234**	Cyclohexyl acetate	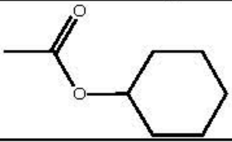

Z	235	Cyclooctyl acetate	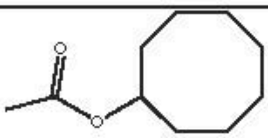
Z	236	Cyclododecyl acetate	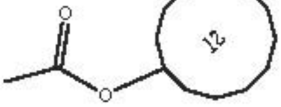
Z	237	Bornyl acetate	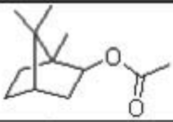
Z	238	Isobornyl acetate	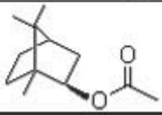
Z	239	Hexyl formate	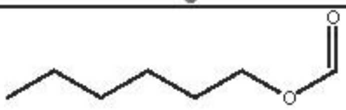
Z	240	Citronellyl formate	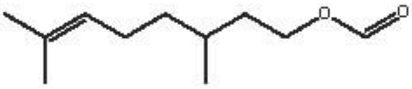
Z	241	Linalyl formate	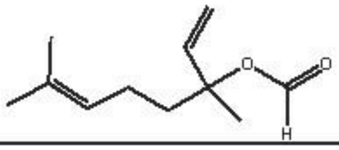
Z	242	-	
Z1	243	-	
Z1	244	Benzyl formate	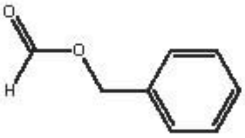
Z1	245	2-phenylethyl formate	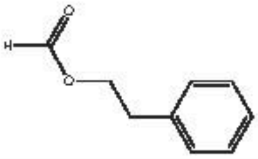

Z1	246	Ethyl benzoate	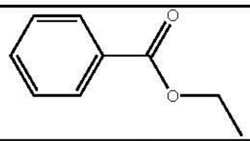
Z1	247	Isobutyl benzoate	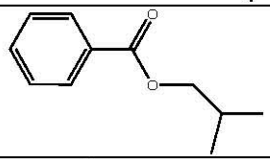
Z1	248	Pipol benzoate	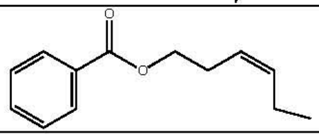
Z1	249	Benzyl benzoate	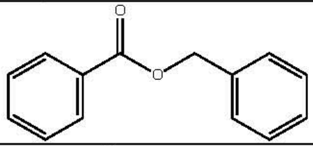
Z1	250	Pipol salicylate	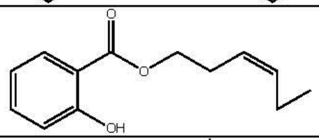
Z1	251	doremox	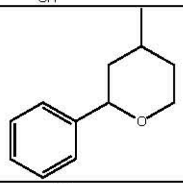
Z1	252	florol	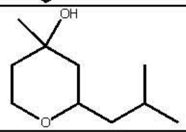
Z2	253	Methyl cyclogeranare	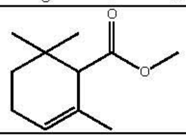
Z2	254	Plicatone	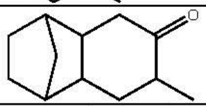
Z2	255	Rhubofix	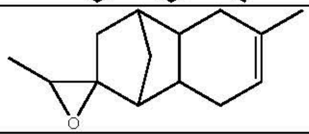
Z2	256	Isopentyrate	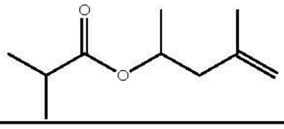

Z2	257	Trifernal	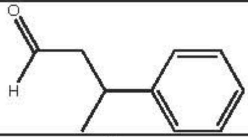
Z2	258	Cyclopidene	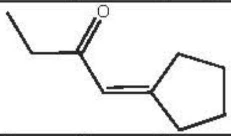
Z2	259	Phenylhexanone	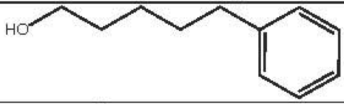
Z2	260	N 431	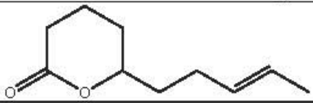
Z2	261	4-undecanolide	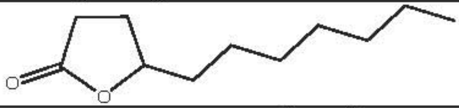
Z2	262	Polysantol	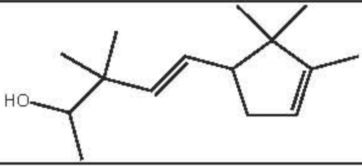
Z3	263	Norlimbanol dextrol	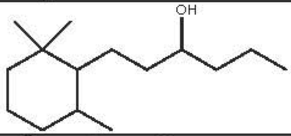
Z3	264	Nootkatone	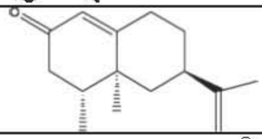
Z3	265	Isononanoique acid	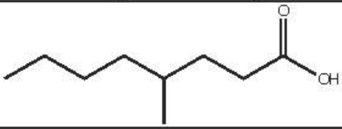
Z3	266	Cedrene epoxide	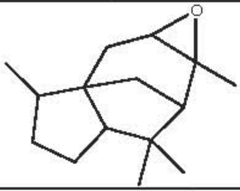
Z3	267	Wolfwood	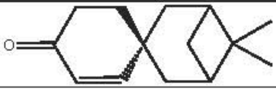

Z3	268	Tricyclone	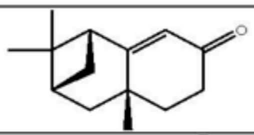
Z3	269	Androstadienone	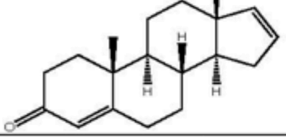
Z3	270	Ethy-L-lactate	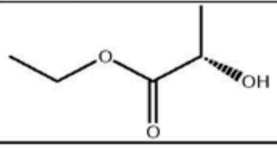

Supplementary list of odorant compounds: Library of odorant molecules used for screening agonists of Olfr74.
